# Selective Suppression of Integrin‐Ligand Binding by Single Molecular Tension Probes Mediates Directional Cell Migration

**DOI:** 10.1002/advs.202306497

**Published:** 2024-02-04

**Authors:** Seong‐Beom Han, Geonhui Lee, Daesan Kim, Jeong‐Ki Kim, In‐San Kim, Hae‐Won Kim, Dong‐Hwee Kim

**Affiliations:** ^1^ KU‐KIST Graduate School of Converging Science and Technology Korea University Seoul 02841 Republic of Korea; ^2^ Biomedical Research Center Korea Institute of Science and Technology Seoul 02792 Republic of Korea; ^3^ Institute of Tissue Regeneration Engineering (ITREN) Dankook University Cheonan 31116 Republic of Korea; ^4^ Department of Biomaterials Science in College of Dentistry & Department of Nanobiomedical Science in Graduate School Dankook University Cheonan 31116 Republic of Korea; ^5^ Department of Integrative Energy Engineering College of Engineering Korea University Seoul 02841 Republic of Korea

**Keywords:** cell adhesion, FAK phosphorylation, integrin α_v_β_3_, molecular tension probes, persistent migration

## Abstract

Cell migration interacting with continuously changing microenvironment, is one of the most essential cellular functions, participating in embryonic development, wound repair, immune response, and cancer metastasis. The migration process is finely tuned by integrin‐mediated binding to ligand molecules. Although numerous biochemical pathways orchestrating cell adhesion and motility are identified, how subcellular forces between the cell and extracellular matrix regulate intracellular signaling for cell migration remains unclear. Here, it is showed that a molecular binding force across integrin subunits determines directional migration by regulating tension‐dependent focal contact formation and focal adhesion kinase phosphorylation. Molecular binding strength between integrin *α_v_β_3_* and fibronectin is precisely manipulated by developing molecular tension probes that control the mechanical tolerance applied to cell‐substrate interfaces. This data reveals that integrin‐mediated molecular binding force reduction suppresses cell spreading and focal adhesion formation, attenuating the focal adhesion kinase (FAK) phosphorylation that regulates the persistence of cell migration. These results further demonstrate that manipulating subcellular binding forces at the molecular level can recapitulate differential cell migration in response to changes of substrate rigidity that determines the physical condition of extracellular microenvironment. Novel insights is provided into the subcellular mechanics behind global mechanical adaptation of the cell to surrounding tissue environments featuring distinct biophysical signatures.

## Graphical abstract

Cell migration is a dynamic cellular response determined by the molecular interaction between integrin and its partner ligands. To regulate the integrin‐mediated signaling, molecular tension probes that control the mechanical tolerance at the integrin‐ligand binding are introduced. This study reveals that manipulation of cell‐ECM adhesion strength at a pico‐newton level can regulate cellular migration by altering intracellular signaling.

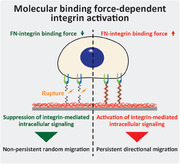

## 1 Introduction

Cell migration is crucial for diverse biological processes such as development, immune response, wound healing, and cancer metastasis.^[^ ^1^ ^]^ During cell migration, cells continuously sense and respond to physical and chemical micro‐environmental alterations, prompting appropriate behavioral changes. Intracellular protein signaling and gene expression regulate cellular migration in response to physical changes in the extracellular environment. The cell adhesion receptor integrin is essential for mediating bidirectional signaling during cellular migration.^[^ ^2^ ^]^ Cells detect external environmental changes and regulate internal cellular responses through this cell membrane receptor. Integrins are heterodimeric receptors comprising alpha and beta subunits that bind to specific ligands in the extracellular matrix (ECM). In the outside‐in integrin signaling pathway, they recruit focal adhesion proteins such as talin, vinculin, paxillin, and focal adhesion kinase.^[^ ^3^ ^]^ These proteins form clusters that serve as signaling hubs and interact with the actin cytoskeleton, inducing cellular forces through actomyosin contractility and transferring intracellular signals to the nucleus.

Cell migration through the integrin pathway is a tightly regulated process requiring precise molecular binding, signaling, and cytoskeletal remodeling coordination. In particular, integrin signaling maturity is predicated by physical endurance of integrin‐ligand interaction, such as ligand density,^[^ ^4^ ^]^ binding force,^[^ ^5^ ^]^ and mobility.^[^ ^6^ ^]^ For instance, sufficient ligand concentrations are required for cluster formation^[^ ^7^ ^]^ when the distance between integrins is less than 30 nm.^[^ ^4^, ^8^ ^]^ Integrin activation also enhances as substrate stiffness increases, with an ≈40 pN binding force.^[^ ^5^, ^9^ ^]^ Because each integrin type contributes distinct cellular forces to sense substrate rigidity,^[^ ^10^ ^]^ these differential binding strengths aid in fine‐tuning cellular responses to mechanical signals.

FAK is a primary downstream effector of integrin signaling events that regulate cell migration, proliferation, and differentiation.^[^ ^11^ ^]^ In the cascade of integrin signaling, FAK is autophosphorylated at tyrosine 397 (Tyr397), opening a high‐affinity binding site for Src‐family kinases.^[^ ^12^ ^]^ The subsequent Src‐mediated FAK phosphorylation at other tyrosine residues amplifies the signal, effectuating numerous adaptor recruitments and signaling proteins to focal adhesions.^[^ ^13^ ^]^ FAK phosphorylation induces the activation of Rho family of small GTPases, including the ras homolog family member A (RhoA), ras‐related C3 botulinum toxin substrate 1 (Rac1), and cell division cycle 42 (Cdc42).^[^ ^14^ ^]^ These molecules regulate actin cytoskeleton dynamics and are integral for cell migration. RhoA promotes actomyosin contractility and stress fiber formation, whereas Rac1 and Cdc42 stimulate lamellipodia and filopodia formation at the leading edge of migrating cells.^[^ ^15^, ^16^ ^]^

Previously, traction force microscopy has been utilized to understand the cellular forces involved in integrin‐mediated cell migration.^[^ ^17^, ^18^ ^]^ However, the sensitivity of this technique is limited to µN and nN scales, restricting exploration into the more delicate molecular changes regulating cellular functions. Therefore, recent advancements have developed molecular tension probes that incorporate integrin ligands and molecular linkers, such as polyethylene glycol (PEG),^[^ ^19^ ^]^ peptide protein,^[^ ^20^ ^]^ and DNA.^[^ ^21^ ^]^ These innovative tools enable real‐time direct measurement of cellular forces exerted at the molecular level with remarkable resolution. For instance, precise programmable DNA structures through sequence changes are primarily used as molecular tension probes because of their malleable molecular binding forces determined by a double‐stranded or single‐stranded DNA backbone.^[^ ^21^ ^]^ DNA‐hairpin molecular tension probe^[^ ^22^ ^]^ and DNA‐origami,^[^ ^23^ ^]^ utilizing twisting and unwinding forces ranging at ≈4 and 19 pN, respectively, rely on single‐stranded DNA. In contrast, tension gauge tethers (TGT) relying on the rupture force of double‐stranded DNA (dsDNA), which is irreversibly broken by cells when the cellular pulling force exceeds the dsDNA binding tolerance, measure 12 to 56 pN depending on the DNA sequence.^[^ ^5^ ^]^

While such molecular tension probes can monitor force‐dependent molecular switching events, intracellular signaling and its related cell motility have yet to be well articulated. Our study explored integrin‐mediated binding force functions in cell migration using a dsDNA‐based tension probe. We crafted a unique surface able to control molecular binding forces by conjugating an integrin‐mediated tension probe to a fibronectin (FN)‐coated substrate. We demonstrated that selectively suppressing integrin‐mediated molecular binding forces can precisely determine focal contact‐guided cell spreading and migration. We investigated how single molecular binding force‐dependent activation of specific integrin subunits regulates FAK phosphorylation. Our results revealed that intracellular signaling, driven by integrin binding forces, regulates global cell migration by reorganization of intracellular polarity. We also established that subtle changes of integrin‐mediated molecular binding force to ECM molecules recapitulate the mechanosensing of ECM rigidity.

## 2 Results

### 2.1 Integrin‐Mediated Molecular Tension Determines Cell Spreading

Subcellular linkage of integrin to ECM components mediates cellular adhesion to extracellular substrates.^[^ ^24^ ^]^ Molecular binding between integrin subunits and ECM ligands is regulated by threshold forces ranging from 40 to 120 pN,^[^ ^25^ ^]^ facilitating intracellular binding to actin filaments via multiple adapter proteins to activate cell‐substrate adhesion‐dependent cellular motion.^[^ ^26^ ^]^ Therefore, we hypothesized that subtle changes of molecular force between integrin subunit and ECM could alter cell migration that requires continuous cell adhesion processes. More specifically, we immobilized molecular tension probes on a FN‐coated surface, where the probe assembly structure is demolished at a determined force, enabling the single molecular force‐dependent selective suppression of integrin binding to ECM.

**Figure 1:**
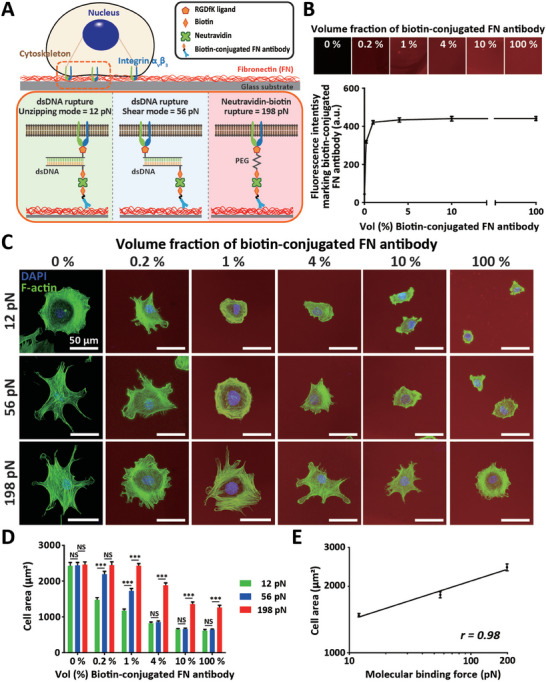
Differential suppression of cell spreading in response to changes in integrin‐mediated ECM binding force. A,B) Modulation of the integrin *α_v_β_3_*‐mediated molecular binding force to ECM via differential molecular tension probes. Schematic representation depicts molecular tension probe‐dependent differential cell adhesion to FN‐coated glass substrates, where tension tolerance of the probes is altered either by switching the RGDfK peptide ligand‐conjugated site in the dsDNA (12 and 56 pN, corresponding to unzipping and shear mode, respectively) or by substituting ligand‐conjugated dsDNA with RGDfK peptide‐conjugated PEG, resulting in rupture of neutravidin and biotin at 198 pN (A). Note that molecular tension probes representing differential nominal rupture forces are immobilized on FN‐coated glass substrates by the biotin‐conjugated FN antibody. Quantitative fluorescence microscopy depicts the rapidly increased fluorescence intensity indicating the amount of biotin‐conjugated FN antibody remained unchanged above 4% of the volume ratio, confirming precisely controlled immobilization of differential molecular tension probes via the biotin‐conjugated FN antibody (B). C–E) Differential changes of cell spreading in response to strength and population of molecular tension probes. Immunofluorescence microscopy of MEFs marking F‐actin (green) and the nucleus (DAPI, blue) visualizes gradual suppression of cell spreading on FN‐coated glass substrates by increasing the population of molecular tension probes from 0% (Control) to 0.2%, 1%, 4%, 10%, and 100% (marked by red), while increasing the integrin *α_v_β_3_*‐mediated molecular binding force from 12 to 56 and 198 pN, enlarges cell spreading area (C). Quantitative analysis of cell spreading depicts significant alteration of cell spreading in response to changes of molecular binding forces among 12 (green), 56 (blue), and 198 pN (red) is detected when 1% (v/v) of biotin‐conjugated FN antibody was applied (D). Under 1% (v/v) of biotin‐conjugated FN antibody used conditions, the cell spreading area and integrin *α_v_β_3_*‐mediated molecular binding force to ECM were tightly related, representing a weak power law, *A_cell_* =  935 · *F* ^0.18^ (E), where A and F indicate cell spreading area and integrin *α_v_β_3_*‐mediated molecular binding force to the substrate, respectively. In panel B, > 40 images were analyzed for each condition. In panels D and E, > 100 cells were analyzed for each condition and statistical differences were calculated using a one‐way ANOVA and Tukey's multiple comparison test. (NS: not significant; ***: *p* < 0.001).

Force‐specific molecular tension probes utilizing rupture forces of dsDNA or biotin‐neutravidin interactions were anchored onto an FN‐coated glass substrate by biotin‐conjugated FN antibodies prior to placing mouse embryonic fibroblasts (MEFs). For cells to attach to the probe, a cyclic (Arg‐Gly‐Asp‐D‐Phd‐Lys) RGDfK peptide was bound to the upper end of the probe, allowing it to selectively bind to integrin *α_v_β_3_*, a major regulatory receptor for early cell adhesion and mechanotransduction^[^ ^27^, ^28^ ^]^ (**Figure** **1**A). dsDNA rupture force‐based molecular tension probes recorded 12 or 56 pN by relocating an RGDfK peptide‐conjugated site in the upper DNA strand, representing a unzipping and shear mode, respectively, in the previously reported tension gauge tether.^[^ ^5^ ^]^ To enhance the mechanical tolerance of the probe via biotin‐neutravidin dissociations, we substituted ligand‐conjugated dsDNA with RGDfK peptide‐conjugated PEG, resulting in 198 pN by measurement with atomic force microscopy (AFM, Figure 1A; Figure S1, Supporting Information). Because molecular tension probes were linked to the FN‐coated glass substrate by biotin‐conjugated FN antibodies, the surface density of immobilized molecular tension probes on the FN‐coated substrate was predicated by biotin‐conjugated FN antibody concentration. To ensure quantitatively precise immobilization of molecular tension probes, we differentiated volumetric concentrations of biotin‐conjugated FN antibodies through phosphate‐buffered saline (PBS) dilution, ranging from 0% (Control, no probes attached) to 0.2%, 1%, 4%, 10%, and 100%. Then, the fluorescence probe DyLight 594 bound to FN antibodies was measured for intensity (Figure 1B). We noted a rapid increase in fluorescence intensity by the augmentation of subtle volumetric content from 0% to 1%, which then remained largely unchanged at 4% asymptotically approaching 100% (Figure 1B). These results substantiate that molecular tension probes were effectively anchored onto the FN‐coated surface by biotin‐conjugated FN antibodies and its optimum concentration for regulating cell adhesion by subtle change of single molecular force can be detected.

To determine optimal experimental conditions for molecular tension‐dependent manipulation of cell adhesion, we monitored the spreading area of MEFs plated on diverse molecular tension probe‐anchored FN‐coated surfaces, where the amount of probes was controlled by the volumetric ratio of probe‐immobilizing biotin‐conjugated FN antibodies (Figure 1C,D). While cell spreading area was enlarged on FN‐coated substrates associated with molecular tension probes exerting greater binding force (12 pN vs 56 pN and 56 pN vs 198 pN), accumulation of molecular tension probes suppressed cell spreading, i.e., increasing biotin‐conjugated FN antibody concentrations from 0% to 100% gradually reduced cell area, regardless of molecular tension probe type (Figure 1C). Systematic analysis of cell area further revealed that cell spreading could be suppressed by reducing the binding force between integrin *α_v_β_3_* and FN, ranging from several tens to hundreds of pico‐newtons (Figure 1D). In molecular tension probe‐absent fully FN‐coated substrates, integrin binds to FN directly without interfering with molecular tension probes, resulting in a maximum cell spreading area (0%, Figure 1D). However, increasing population of molecular tension probes controlled by biotin‐conjugated FN antibody concentrations differentially reduced cell spreading in response to changes of integrin‐mediated molecular force. For instance, low binding tolerance (i.e., 12 and 56 pN) significantly reduced the cell spreading area even at relatively low concentration of molecular tension probes (0.2% and 1%, Figure 1D), where a majority of integrin subunits still directly bind to FN and high binding tolerance (i.e., 198 pN) did not noticeably reduce cell spreading from bare FN substrates. In contrast, at enhanced population of molecular tension probes (4%, 10%, and 100%, Figure 1D), substituting direct integrin binding to FN with a 198 pN‐tension probe suppressed cell spreading, and a half‐fold change was indicated at above 10% of the population, while cells plated on the low force tolerance probe‐anchored substrates (i.e., 12 and 56 pN) largely maintained their reduced spreading area without further changes (Figure 1D). Moreover, we noted that significant differences in cell spreading areas due to changes of molecular binding tolerance among 12, 56, and 198 pN were detected only at 1% of the molecular tension probe population (Figure 1D). Because cells were rarely adhered to the the surface that was coated with 100% biotin‐conjugated FN antibody without molecular tension probes (Figure S2, Supporting Information), these results further confirms that 1% of the molecular tension probe population is the optimal condition to tightly regulate the molecular binding force‐dependent modulation of cell adhesion.

To verify if differential cell spreading at a determined surface density of molecular tension probes was purely regulated by structural distinction of our designed probes, we investigated whether limited cell adhesion from reduced integrin‐mediated molecular tension could be recovered only by disrupting dsDNA strands (Figure S3, Supporting Information). Treatment of cell‐anchored surfaces with DNase I, a nuclease enzyme that preferentially digests DNA strands at phosphodiester bonds 5′ to pyrimidine nucleotides,^[^ ^29^ ^]^ selectively removed DNA rupture force‐based molecular tension probes without interfering with biotin‐neutravidin interactions (Figure S3A, Supporting Information). Therefore, only the spreading area of cells placed on DNA‐based molecular tension probe‐coated surfaces (i.e., 12 and 56 pN‐probes) was recovered to values of cells placed on biotin‐neutravidin dissociation‐based molecular tension probe‐coated surfaces (i.e., 198 pN‐probe) (Figure S3B, Supporting Information). Because cells placed on 198 pN‐surfaces exhibited the same spreading area as cells placed on probe‐absent control FN‐coated substrates, these results confirm that DNA rupture force‐based molecular tension probes selectively suppress cell adhesion by fine‐tuning the integrin‐mediated single molecular force. These results further suggest that strong molecular interactions approximating the biotin‐neutravidin binding interaction do not interfere with integrin‐mediated cell adhesion to FN.

A previous report demonstrated that differential cell spreading in response to substrate stiffness changes followed power laws, enabling the detection of effective substrate elasticity to control cell adhesion. Therefore, we asked if the alteration of binding force at a single molecular level between small amounts of integrin subunits in an adherent cell and the ECM could also precisely regulate cell adhesion. By replotting the cell area to the strength of single molecular binding force in a determined population of molecular tension probes with a log‐log scale, we noted a tight correlation representing *A*  =  935 · *F* ^0.18^, where A and F represent cell area and molecular binding force between a single integrin *α_v_β_3_* subunit and FN, respectively (Figure 1E).

Together, these results demonstrate that fractional reduction of the pN‐scaled molecular binding force between an integrin subunit and FN can effectively suppress cell adhesion.

### 2.2 Integrin‐Mediated Ligand Binding Force Determines Cell Adhesion through Focal Contact between Cell and ECM

**Figure 2:**
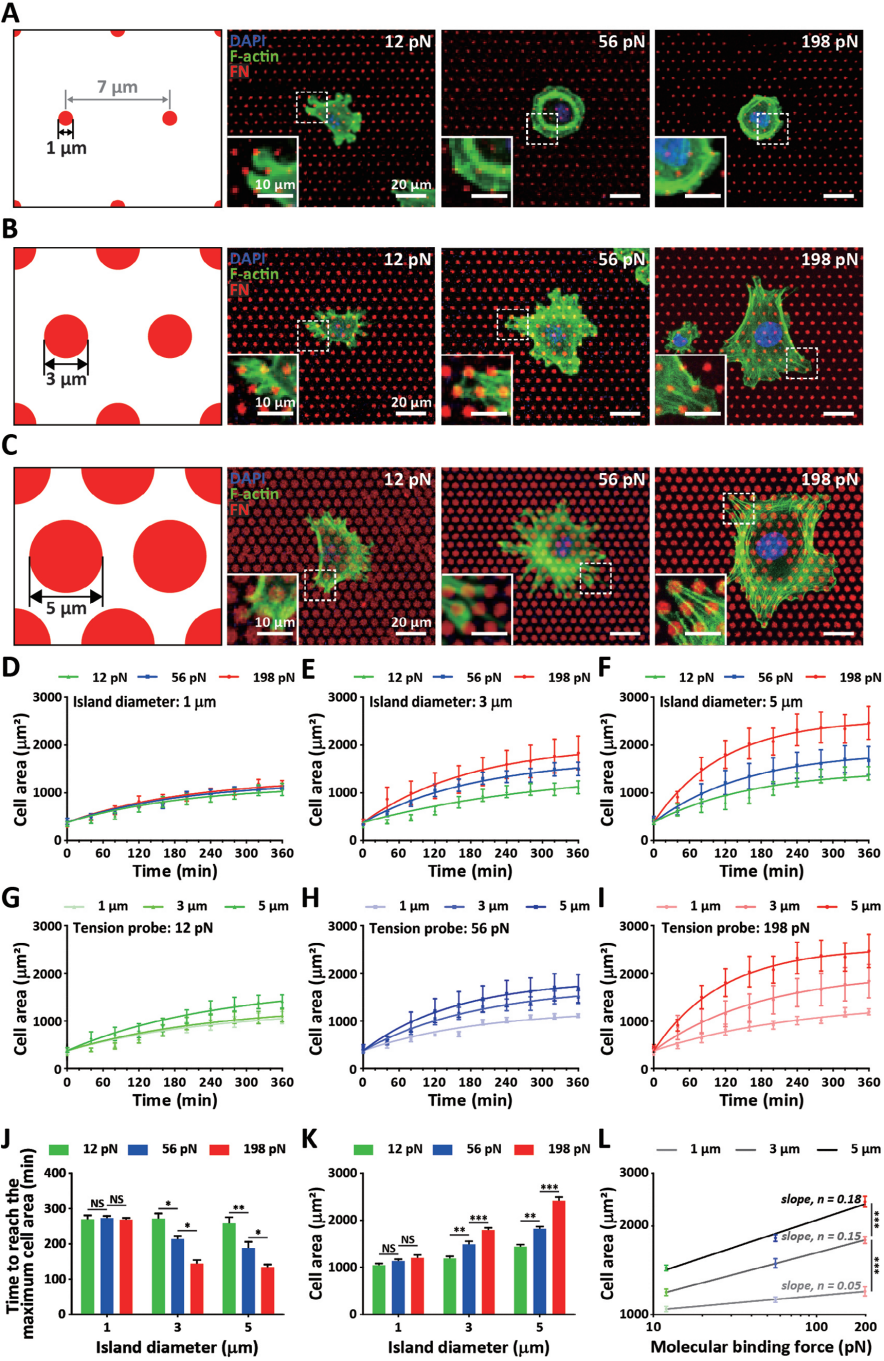
Modulation of cell spreading in response to differential surface coverage of FN‐anchored molecular tension probes. A–C) Alteration of cell spreading in response to integrin *α_v_β_3_*‐mediated molecular binding forces in a confined cell‐ECM contact. MEFs placed on size‐controlled circular islands filled with differential molecular tension probe‐conjugated FNs (red, denoted by 12, 56, and 198 pN) were immuno‐stained to visualize the nucleus (DAPI, blue) and F‐actin (green), where diameters of FN circular island were 1, 3, and 5 µm, respectively. D–I) Time‐dependent monitoring of cell spreading in response to molecular binding strength and cell‐ECM contact size. The spreading area of MEFs placed on substrates with varying diameter of FN‐circular islands (1 (D), 3 (E), and 5 µm (F)) conjugated with molecular tension probes gauging 12 (G), 56 (H), and 198 pN (I), respectively, was captured every 20 min for 6 h. Cell spreading area rapidly increased following the initial cell adhesion to FN islands but remained largely unchanged, which is more prevalent in cells placed on larger FN islands (D–F), resulting in a more pronounced cell spreading area at the end of time‐lapse monitoring (G–I). Note that time‐dependent increase of cell spreading area follows a typical logarithmic growth curve with distinct saturation points (D–I). J–L) Differential cell spreading dynamics in response to partial changes of integrin *α_v_β_3_*‐mediated molecular binding force within controlled cell‐ECM contact. Increasing integrin *α_v_β_3_* binding strength to FN by molecular tension probes accelerated cell spreading in 3 µm‐ and 5 µm‐sized cell‐ECM contacts, reducing the saturation time for cell spreading (J) and resulting in greater cell areas (K), while no changes were observed regarding 1 µm‐sized cell‐ECM contact (J,K). Linear regression on the log‐log scale depicts a more substantial cell spreading dependence on molecular tension probe strength in enlarged cell‐ECM contacts (L). The slope of linear regression represents the exponent value *n* in *A_cell_* =  *Const* · *F^n^*, where *n* indicates the cell enlargement rate by increasing molecular binding force. In panels D‐L, > 30 cells were analyzed for each condition. Error bars indicate S.E.M. Statistical differences were calculated using one‐way ANOVA and Tukey's multiple comparison test. (NS: not significant; *: *p* < 0.01; **: *p* < 0.005; ***: *p* < 0.001).

Cell adhesion to the substrate accompanied by the integrin‐mediated ligand binding is tightly regulated by cell‐ECM contact, where focal adhesion organizations precisely control cell spreading dynamics that determines cell motility via establishment of cell polarity.^[^ ^30^, ^31^ ^]^ To further determine if alteration of cell adhesion in response to binding force changes between the integrin subunit and ECM at a single molecular level was also mediated by focal contact between cell and ECM, we manipulated the dimension of cell‐ECM contact site, which could differentiate the level of integrin binding force to the ECM. To this end, we first designed microcontact‐printed microarrays of FN islands separated by cell‐adhesion repelling poly(L‐lysine)‐graft‐poly(ethylene glycol), where island size was systematically enlarged from 1 to 3 and 5 µm in diameter (**Figure** **2**A–C; and Movies S1–S3, Supporting Information), analogous to focal adhesion length scales detected in randomly migrating MEFs.^[^ ^30^ ^]^ Prior to placing cells, FN islands were partially conjugated with molecular tension probes measuring 12, 56, and 198 pN, as previously determined (Figure 1E). We noted that cell protrusions marked by F‐actin staining were primarily detected at the periphery of the cell located on large FN‐islands conjugated with potent molecular tension probes (*insets*, Figure 2A–C), suggesting that integrin‐mediated molecular tension alters cell adhesion through the focal contact at the cell‐ECM interface.

Time‐lapse monitoring of the cell spreading area on molecular tension probe‐conjugated microarrays of FN islands predominantly displayed logarithmic growth featuring a distinct saturation point depending on the size of focal contact (Figure 2D–F) and integrin *α_v_β_3_*‐mediated molecular binding forces (Figure 2G–I). Consistent with previous results indicating that increasing focal contact area accelerated cell adhesion,^[^ ^31^ ^]^ cells placed on our molecular tension probe‐conjugated FN microarrays reached their maximum spreading area faster in 5 µm‐FN islands than cells placed on 1 and 3 µm‐FN islands (Figure 2D–F). We also noted that this focal contact size‐dependent shift in cell spreading dynamics was most prominent in 198 pN‐probes conjugated islands and abrogated by reducing the integrin‐mediated molecular tension (Figure 2G–I)., I.e., the time to reach the maximum spreading area available in each condition, denoted by saturation time, was 260 min in 1 µm‐FN islands, regardless of the molecular tension probes, which was reduced by strengthening molecular binding force from 12 to 56 and 198 pN in 3 or 5 µm‐molecular tension probe‐conjugated microarrays (Figure 2J). Because focal contact‐mediated cell adhesion is highly sensitive to the initial cell spreading,^[^ ^31^ ^]^ reduced saturation time (i.e., accelerated cell adhesion) results in enhanced cell spreading areas and vice versa (Figure 2K).

To further confirm the significance of size‐dependent focal contact, we monitored the cell spreading dynamics on the molecular tension probe‐conjugated FN microarrays maintaining a constant gap distance between FN islands (Figure S4, Supporting Information). As expected, cell spreading kinetics was reduced on this micropattern, i.e., time to reach the maximum cell area was delayed, because surface coverage of FN was reduced (Figure 2A–I vs Figure S4A–I, Supporting Information). However, consistent with previous results, overall trend of cell spreading dynamics was maintained in both cases, i.e., the time to reach the maximum spreading area was reduced and the final cell spreading area was increased by enlarging the FN island size from 1 to 3 and 5 µm (Figure 2J, K vs Figure S4J,K, Supporting Information). These results reconfirm that dimension of focal contact is critical to determine the cell spreading dynamics.

Interestingly, we noted that the cell spreading rate was independent of molecular binding forces in a relatively small focal contact (i.e., in the 1 µm‐FN island microarray) (Figure 2D,J,K). Together with previous results demonstrating that integrin *α_v_β_3_*‐FN binding force‐dependent cell spreading changes were intensified by increasing the surface density of molecular tension probes from 0 to 1% (Figure 1D), this result highly suggests that a threshold of the molecular binding interaction between an integrin subunit and ECM is required to alter cell‐to‐substrate adhesion. As tested in the unconfined FN surface (Figure 1E), linearity between cell spreading area and molecular binding force in the log‐log scale also followed the power law, formulated as *Cell* *area*  =  *Const* · (*Molecular* *binding* *force*)^*n*^, featuring distinct exponent values *n*, depending on focal contact size (Figure 2L). We found that exponent values *n* increased from 0.05 to 0.15 and 0.18 by enlarging the diameter of FN islands from 1 to 3 and 5 µm, respectively (Figure 2L). These results indicated that molecular binding force‐dependent cell spreading was more sensitive on the 5µm‐FN islands (*n* = 0.18) than on the 1µm‐FN islands (*n* = 0.05). We further noted that the exponent value *n* on 5µm‐FN islands was the same as that in a fixed population of molecular tension probes conjugated on the unconfined FN surface (*n* = 0.18, Figure 1E). Accordingly, we observed almost identical alteration of cell spreading in response to changes of molecular binding force on a fully FN‐coated surface (Figure 1D) and 5 µm‐FN islands (Figure 2K).

These results suggest a subtle shift in molecular interaction between an integrin subunit and ECM could modulate cell adhesion by transferring binding forces through focal contact.

### 2.3 Integrin‐Mediated Differential Ligand Binding Force Modulates Focal Adhesion Formation

**Figure 3:**
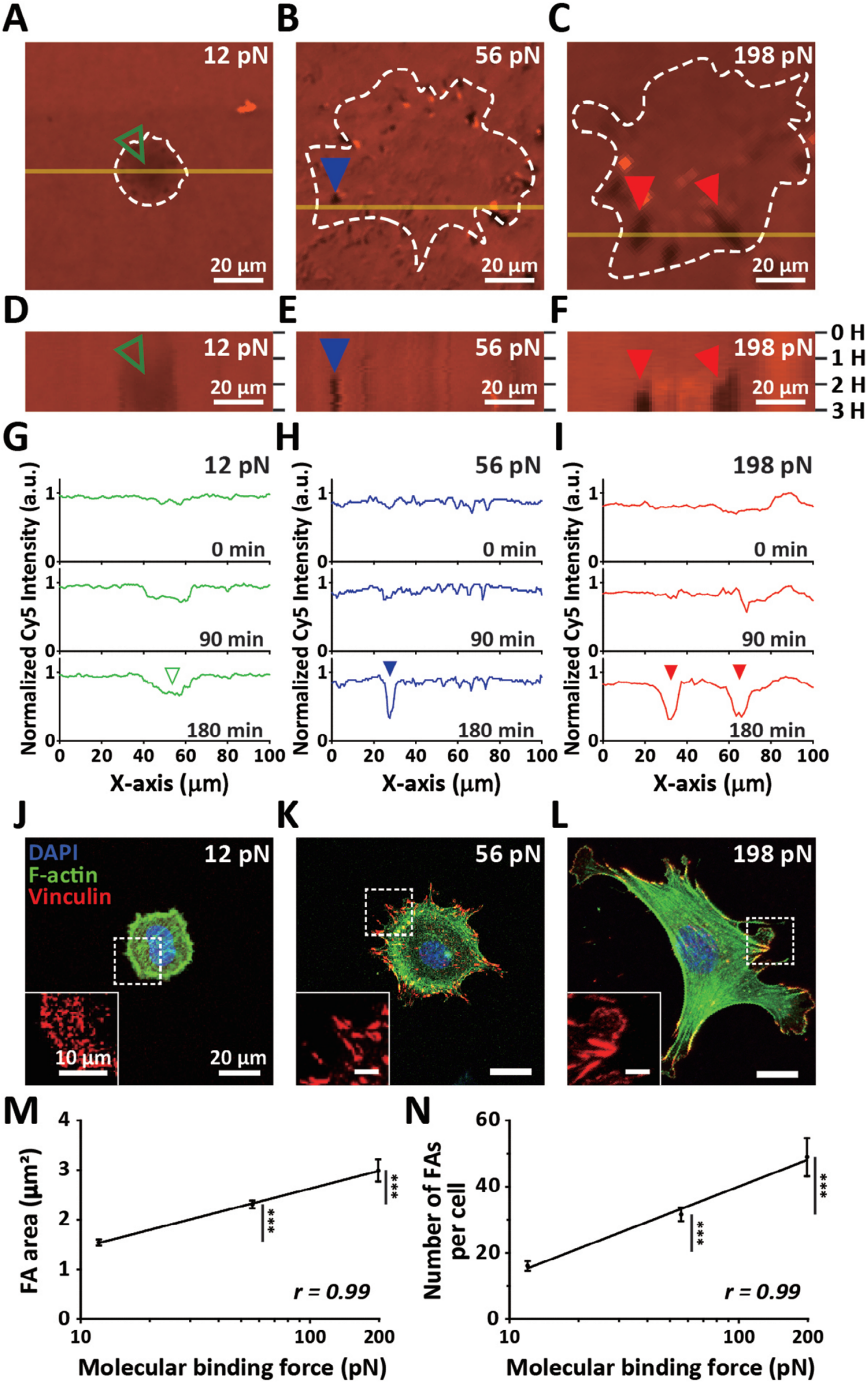
Focal adhesion‐dependent differential force transmission via molecular tension probes. A–I) Live cell monitoring of focal adhesion‐mediated force transmission. Subcellular force surpassing the molecular binding force between integrin *α_v_β_3_* and FN‐coated substrates ruptured molecular tension probes, forming dark spots indicating the dissociation of fluorescence molecule Cy5 (far red) bound to upper ssDNA (12 pN and 56 pN‐probes) or PEG (198 pN‐probe) from molecular tension probe‐engaged FN‐coated surfaces (A–C). Note that more substantial molecular binding forces leave traces of fluorescence loss closer to the periphery of the cell (white dashed lines). The largest defects along the cell boundary were observed in 198 pN‐probe‐conjugated FN‐coated surfaces (red arrowheads), where full and empty arrowheads indicate the presence and absence of rupture traces, respectively (A–C). Representative kymographs crossing the cell body (yellow solid lines, A–C) show time‐dependent changes of fluorescence intensity profiles, where dark regions indicate the location of ruptured molecular tension probes gradually growing throughout the cell (D) or specifically localized along cell boundaries (E,F). Note that relatively more localized dark regions were detected in 198 pN‐probe‐conjugated FN‐coated surfaces than in 56 pN‐probe‐conjugated FN surface, which were widened over time (D vs E, F). Kymographs were recorded every 5 min for 6 h (D–F). Normalized fluorescence intensity profiles obtained from three different time points (0, 90, and 180 min) were compared in each molecular tension probe‐engaged FN surface (G–I). Notably, 12 pN‐probes did not exhibit a localized drop in fluorescence intensity (G), but sharp decline of fluorescence intensity (H,I) and their widening (I) were captured in 56 pN and 198 pN‐probes engaged FN surfaces. J–N) Differential formation of focal adhesions in response to changes of molecular binding force across integrin *α_v_β_3_*. Vinculin‐stained (red) MEFs were co‐stained for nucleus (DAPI, blue) and F‐actin (green) following culturing on differential molecular tension probe‐engaged FN‐coated substrates for 3 h (J–L). *Insets* depict the detailed organization of focal adhesions marked by vinculin. Note that diffused punctate vinculin staining changed to clustered focal adhesions specifically localized along the cell boundary by increasing molecular binding force (12 pN vs 56 and 198 pN). Conjugation of molecular tension probes representing elevated molecular binding force between integrin subunits and FN‐coated substrates significantly increased size (M) and number (N) of focal adhesions per cell. Strong correlations between molecular binding force and focal adhesion formation were determined by high Pearson correlation coefficient *r* values (0.99, M and N). In panels M and N, >30 cells were analyzed for each condition. Error bars indicate S.E.M. and statistical differences were calculated using one‐way ANOVA and Tukey's multiple comparison test. (***: *p* < 0.001).

We demonstrated that cell spreading dynamics was highly sensitive to the cell‐ECM contact dimension,^[^ ^31^ ^]^ where increasing the molecular binding force between an integrin subunit and its ligand partner accelerated cell spreading (Figure 2). Moreover, blocking actomyosin contractility disrupted force transmission between focal adhesion and the underlying substrate.^[^ ^32^ ^]^ Thus, we hypothesized that differential focal adhesion formation induced molecular binding force‐dependent alteration of cell spreading. To determine whether integrin‐ligand binding force could alter force transmission to the substrate through focal adhesions, we monitored fluorescence intensity of Cy5 conjugated on molecular tension probes (**Figure** **3**A–I; and Movie S4, Supporting Information). The Cy5 rupture trace indicated the region where the subcellular pulling force was greater than the applied mechanical tolerance of molecular tension probes^[^ ^5^ ^]^ (Figure S5, Supporting Information). Although cells incubated for 3 h on the 12 pN‐probe‐engaged FN surfaces weakly reduced fluorescence intensity in the entire cell area (Figure 3A), cells plated on 56 or 198 pN‐probe‐engaged FN surfaces displayed distinct regions indicating the breakage of molecular tension probes along the cell periphery, where enlarged rupture traces were detected on 198 pN‐probe‐engaged FN surfaces (Figure 3B,C). Cy5 intensity kymographs at the region of fluorescence loss further revealed distinct formation of rupture traces (Figure 3D–I). While slightly reduced fluorescence intensity was maintained underneath the whole cell area on 12 pN‐probes (Figure 3D,G), more focal adhesion‐like traces of fluorescence loss were detected on 198 pN‐probes than on 56 pN‐probes, and their width increased over time (Figure 3E,H vs F,I).

Next, to confirm that integrin‐mediated submolecular binding force regulates formation of focal adhesions, we quantified the size and number of focal adhesions in vinculin‐immuno‐stained cells lying on differential tension probes‐conjugated FN surfaces (Figure 3J–L). Compared to the punctate distribution of vinculin‐marked focal adhesions in cells on 12 pN‐probes, enlarged focal adhesion clusters were detected by increasing integrin‐mediated molecular binding strength (Figure 3J vs 3K,L). Consequently, strong correlations between integrin‐mediated molecular binding force and the size (Figure 3M) or number (Figure 3N) of focal adhesions were determined. This data reveal that focal contact‐dependent changes of cell spreading is attributed to the differential formation of focal adhesion that is tightly regulated by integrin‐mediated ligand binding force (Figures 2 and 3J–N).

Together with the previous results indicating that matured focal adhesions exert more substantial pulling force on cell‐bound substrates^[^ ^,33^ ^]^ these results highly suggested that greater integrin‐mediated molecular binding force could mature focal adhesions, which in turn, prompting at cell‐substrate interface.

### 2.4 Integrin‐Mediated Ligand Binding Force Regulates FAK Phosphorylation

Accumulation of integrin receptor proteins and their binding to ECM ligand molecules promote activation of FAK,^[^ ^34^ ^]^ where FAK dimerization induces auto‐phosphorylation at Tyr397 with Src.^[^ ^12^ ^]^ Overexpressed FAK, acting as a major integrin‐mediated intracellular signaling kinase in ovarian^[^ ^35^ ^]^ and breast cancers,^[^ ^36^ ^]^ is attributed to FAK‐dependent invasion and metastatic potential.^[^ ^37^ ^]^ As previous results confirmed that regulation of cell spreading by micropatterning altered intracellular cytoskeletal tension,^[^ ^38^ ^]^ cell‐ECM binding force modulated cell spreading (Figure 1). The integrin‐mediated differential molecular binding force maintained relative FAK expression per cell constant (Figure S6, Supporting Information), and focal adhesion size determined the expression of phosphorylated FAK.^[^ ^39^ ^]^ Thus, we hypothesized that subtle changes in molecular binding force between integrin and ECM could regulate FAK activation.

**Figure 4:**
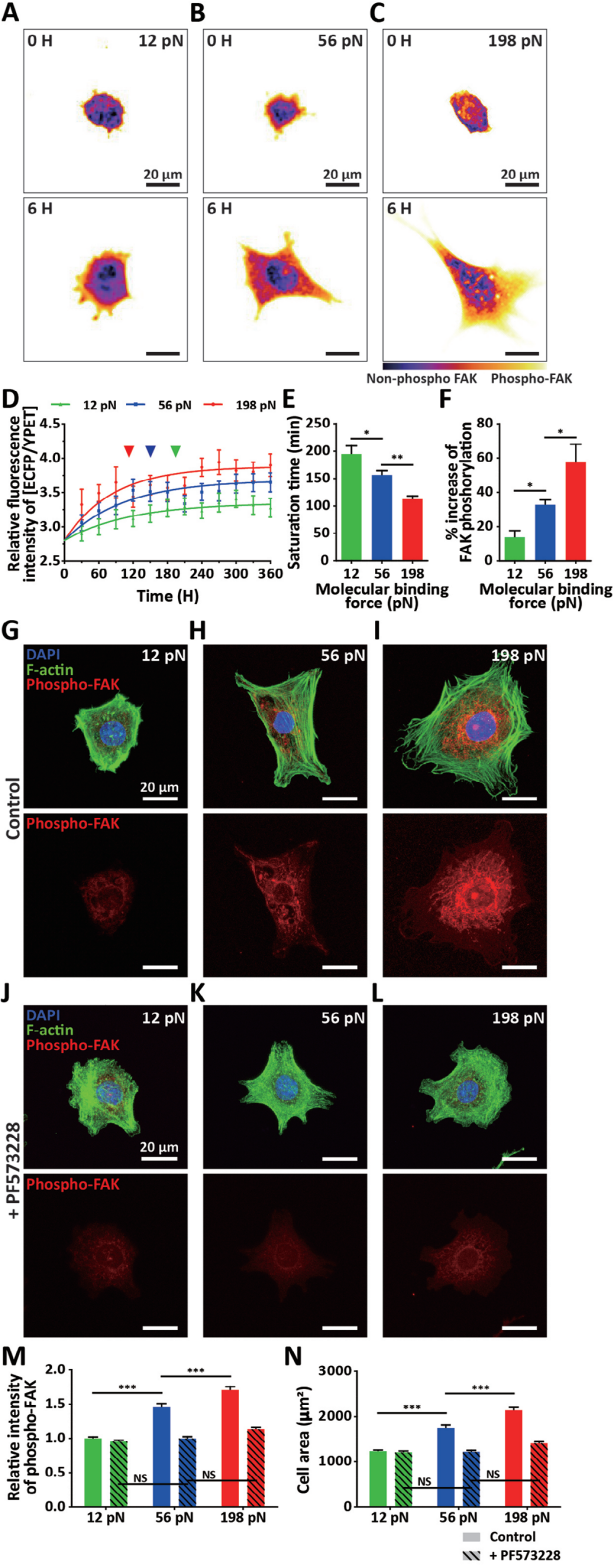
Differential activation of intracellular signaling in response to integrin‐ligand binding force. A–F) Altered FRET signal of FAK phosphorylation in response to integrin‐ligand binding strength. Representative FRET signals depict FAK phosphorylation in FRET biosensor‐transfected MEFs placed on FN‐coated substrates conjugated with differential molecular tension probes gauging 12 (A), 56 (B), and 198 pN (C); purple and yellow indicate low and high levels of FAK phosphorylation, respectively. Note that enlarged cells in 198 pN‐probe‐conjugated substrates reveal enhanced FAK phosphorylation. Time‐lapse monitoring of relative fluorescence intensity between ECFP and YPET (denoted by [ECFP/YPET]) indicated FRET efficiency for FAK phosphorylation, performed every 30 min for 6 h to visualize molecular binding force‐dependent differential FAK phosphorylation (D). Increasing molecular binding force accelerated FAK phosphorylation, significantly reducing saturation time (D,E) and significantly increasing FAK phosphorylation compared to non‐phosphorylated FAK (F). G–N) Confirming integrin‐mediated molecular binding force‐dependent FAK phosphorylation. MEFs placed on FN‐coated substrates conjugated with differential molecular tension probes were immuno‐stained for phosphorylated FAK (red), nuclear DNA (DAPI, blue), and F‐actin (green) after 3 h of cell plating with/without treatment of FAK‐phosphorylation inhibitor (PF573228) (G–L). Inhibiting FAK phosphorylation abrogated molecular binding force‐dependent alteration of FAK phosphorylation and cell spreading areas compared to the control, where enlarged cells by increasing molecular binding force depicted enhanced FAK phosphorylation (G–I vs J–L). Quantitative analysis indicated that fluorescence intensity of phosphorylated FAK (M) and cell spreading area (N) significantly increased in response to integrin *α_v_β_3_*‐mediated ligand binding strength, which was abrogated by inhibiting FAK phosphorylation (solid bars vs dashed bars). In panels D‐F, > 20 cells were analyzed for each condition. In panels M and N, > 200 cells were analyzed for each condition. Error bars indicate S.E.M. and statistical differences were calculated using one‐way ANOVA and Tukey's multiple comparison test. (NS: not significant; *: *p* < 0.01; **: *p* < 0.005; ***: *p* < 0.001).

To determine if integrin‐mediated force transmission could regulate the activation of intracellular signaling, we monitored spatiotemporal changes in Förster resonance energy transfer (FRET) signals induced by auto‐phosphorylation at Tyr397 (**Figure** **4**A–F). We utilized a previously developed FAK FRET biosensor consisting of enhanced cyan fluorescent protein (ECFP)‐conjugated Src homology 2 (SH2) domain, a yellow fluorescent protein YPet‐conjugated Tyr397‐encompassing substrate sequence, and their flexible linker peptides, where FAK phosphorylation at Tyr397 induces SH2 domain binding, resulting in the increased distance between fluorescence proteins.^[^ ^40^ ^]^ Because FAK phosphorylation‐induced reduction of FRET efficiency is determined by the enhanced ECFP/YPet emission ratio, molecular binding force‐dependent changes of FAK phosphorylation were differentially color‐coded; purple and yellow indicated low and high FAK phosphorylation levels, respectively (Figure 4A–C; and Movie S5, Supporting Information). We noted that low FAK phosphorylation levels were maintained on 12 pN‐probe‐conjugated FN surfaces (Figure 4A), but enhanced FRET signals (i.e., increased FAK phosphorylation) were detected after 6 h along the periphery of cells placed on FN surfaces conjugated with 56 or 198 pN‐probes (Figure 4B,C). Time‐lapse monitoring of relative ECFP and YPet emission intensities further indicated that the magnitude and rate of FAK phosphorylation were highly sensitive to integrin‐mediated molecular binding force (Figure 4D), where increasing the integrin‐ligand binding force accelerated FRET transfer (Figure 4E), resulting in the enhanced FAK phosphorylation, and vice versa (Figure 4F).

Because differential FAK phosphorylation in response to integrin‐mediated molecular binding force was consistent with cell spreading changes (Figure 2), we investigated whether integrin‐ligand binding force‐dependent FAK phosphorylation could directly regulate cell spreading. To test this notion, we quantified cell spreading areas after disrupting FAK phosphorylation by treating cells plated on the differential molecular tension probes with PF573228, an pharmaceutical inhibitor of phosphorylation at Tyr397 (Figure 4G–N).^[^ ^41^ ^]^ Compared to dimethyl sulfoxide (DMSO)‐treated control conditions exhibiting molecular binding force‐dependent FAK phosphorylation (Figure 4G–I), PF573228 treatment suppressed phosphorylated FAK expression (Figure 4J–L), resulting in no significant responses to differential molecular tension probes (Figure 4M). Consequently, integrin‐mediated molecular binding force‐dependent differential cell spreading in control conditions was abolished by inhibiting FAK phosphorylation (Figure 4N). We further noted that 10 µm of PF573228, not having critical impacts on cellular signaling pathways such as apoptosis and the cell cycle (Figure S7, Supporting Information), recovered the enhanced FAK phosphorylation and cell spreading by increasing molecular binding forces (i.e., 56 or 198 pN) to those of control cells plated on FN surfaces conjugated with 12 pN‐probes (Figure 4M,N).

Combined with the results indicating a similar FAK expression across cells on differential molecular tension probes (Figure S6, Supporting Information) and molecular binding force‐dependent formation of focal adhesions (Figure 3), these results demonstrate that the alteration of cell adhesion in response to integrin‐ligand binding force is precisely controlled by FAK phosphorylation, not by total amount of FAK. This observation further implies that molecular binding strength between integrin subunits and ECM ligands can regulate the activation of intracellular signaling to modulate cell adhesion to the ECM.

### 2.5 Integrin‐Mediated Molecular Binding Force Modulates Persistent Cell Migration via FAK Phosphorylation

Autophosphorylation of FAK at Tyr397 enhances not only the maturation of focal adhesions but turnover rate of clustering at the front end of translocating cells,^[^ ^42^ ^]^ which ultimately regulates the directional cell migration.^[^ ^43^ ^]^ Because we found that subtle changes in the molecular binding force of integrin subunit to ECM ligands altered focal adhesion‐dependent cell adhesion strength (Figures 1, 2, 3) and intracellular signaling of FAK activation (Figure 4), we hypothesized that the integrin‐mediated molecular binding force could regulate persistent cell migration via FAK phosphorylation.

**Figure 5:**
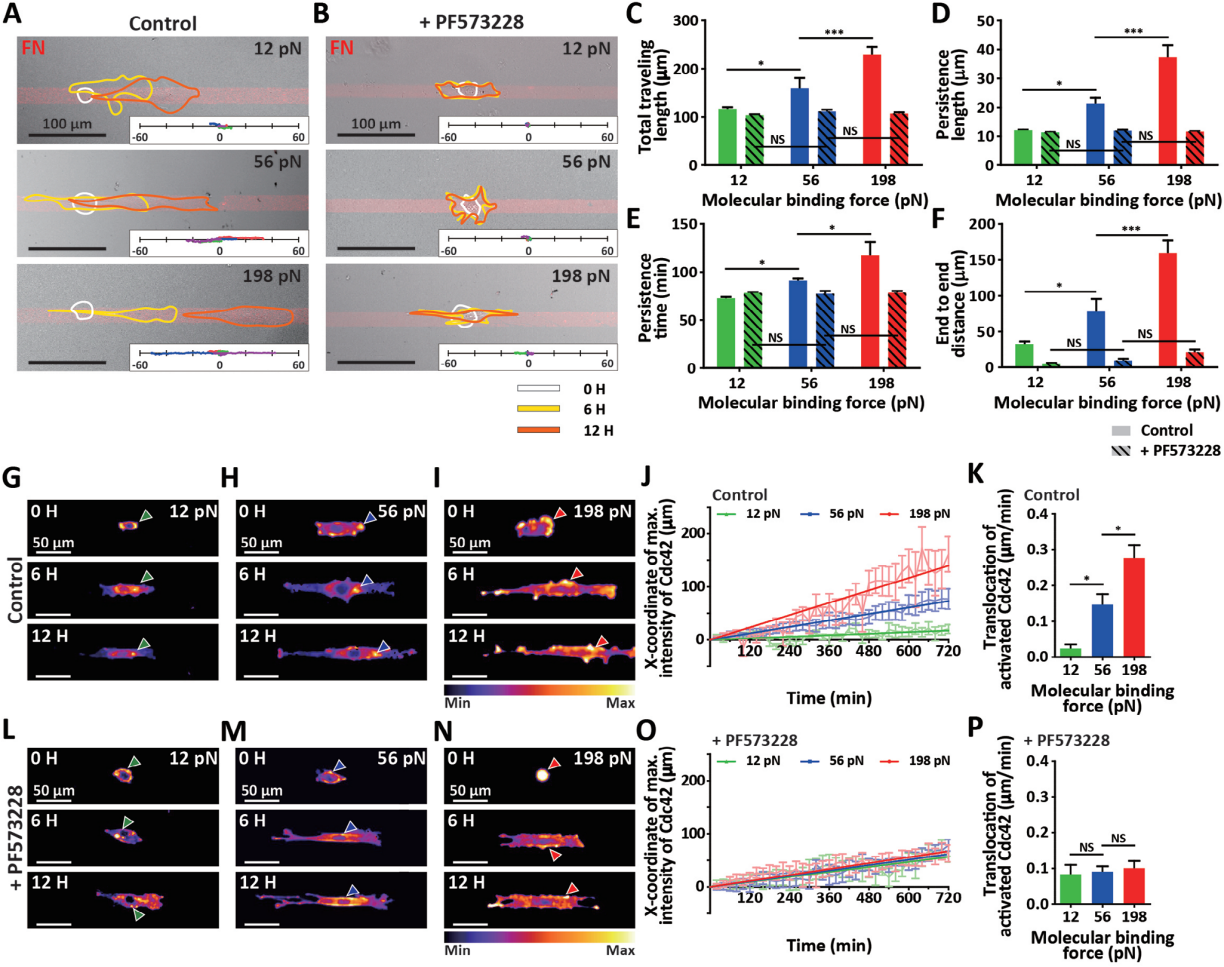
Modulation of directional cell migration via integrin‐ligand binding force‐dependent FAK phosphorylation. A–F) Suppression of molecular binding force‐dependent directional cell migration by inhibiting FAK phosphorylation. Time‐lapse monitoring of migrating cells confined on a 20 µm stripe micro‐patterned FN (red) selectively conjugated with molecular tension probes gauging 12, 56, and 198 pN with/without treatment of FAK phosphorylation inhibitor (PF573228) (A,B). White, yellow, and orange lines indicate cell boundaries captured at 0, 6, and 12 h, respectively. *Insets* depict representative cell trajectories for each condition. Note that cells were allowed to translocate along the FN‐stripe patterns due to cell‐adhesion‐repelling chemical passivation beyond the FN‐coated region. Quantitative analysis of cell motility indicated that increasing integrin‐mediated molecular binding force enhanced total traveling length (C) and persistent motion (D,E), resulting in more directional movement (F), which is abrogated by inhibition of FAK phosphorylation (solid bars vs dashed bars, C–F). G–P) Inhibition of cell polarity during molecular binding force‐dependent directional cell migration by inhibition of FAK phosphorylation. Spatiotemporal monitoring of Cdc42 activation in persistently migrating MEFs on micro‐patterned FN stripes engaged with integrin‐mediated molecular tension probes gauging 12, 56, and 198 pN was performed in control (G–K) and FAK phosphorylation inhibitor PF573228‐treated conditions (L‐P). Representative images were captured at 0, 6, and 12 h; green, blue, and red arrowheads indicate the position showing the maximum intensity of activated Cdc42; purple and yellow indicate low and high levels of Cdc42 activation, respectively (G–I and L–N). Plotting the x‐coordinates indicating the highest intensity of activated Cdc42 with monitoring time revealed the enhanced translocation by increasing integrin‐mediated molecular binding forces (J, K), which was abrogated by inhibiting FAK phosphorylation (O,P). In panels C‐F, > 20 cells were analyzed for each condition. In panels J, K, O, and P, > 15 cells were analyzed for each condition. Error bars indicate S.E.M.; statistical differences were calculated using one‐way ANOVA and Tukey's multiple comparison test. (NS: not significant; *: *p* < 0.01; ***: *p* < 0.001).

To systematically assess the molecular binding force‐dependent migratory persistence, we designed cell adhesive micropatterns of differential molecular tension probe‐engaged FN stripes with a 20 µm width, where cellular motion was monitored for 12 h in DMSO‐treated control (**Figure** **5**A) or FAK phosphorylation‐inhibiting PF573228‐treated conditions (Figure 5B). While stripe‐FN‐micropatterns specifically allowed cell elongation, compared to control cells displaying directional cell motion along the narrow cell‐adhesive FN region, FAK phosphorylation‐inhibited cells did not translocate (Figure 5A,B; and Movie S6, Supporting Information). Analyzing persistence vectors obtained by tracking cell centroids confirmed that control cells tended to maintain their persistent movement by increasing the integrin‐mediated ligand binding force without switching their migratory modes, which was abrogated by inhibiting FAK phosphorylation (Figure 5C). Accordingly, in contrast to control cells showing significantly enhanced persistent migration by increasing the integrin‐mediated molecular binding force, inhibition of FAK phosphorylation suppressed persistent cell movement (Figure 5D–F). We further noted that FAK de‐phosphorylation not only suppressed persistent directional motion but eliminated integrin‐ligand binding force‐dependent differences (Figure 5C–F). These findings suggest that FAK phosphorylation‐dependent persistent cell migration was highly sensitive to integrin subunit‐ligand binding strength.

Previously, we demonstrated that cell polarity during the initial cell adhesion to the substrate was tightly regulated by dynamic rearrangement of cytoskeleton proteins via intracellular signal pathways incorporating small Rho family GTPases. In addition, the intracellular localization of activated Cdc42 determined the cell polarization to initiate directional cell migration.^[^ ^31^ ^]^ Therefore, we investigated whether FAK phosphorylation could regulate activated Cdc42 positioning to confirm that integrin‐mediated molecular binding force‐dependent persistent cell migration was induced by phosphorylated FAK signaling. Specifically, we monitored spatiotemporal localization of activated Cdc42, where a Raichu‐Cdc42 FRET probe and Ras interaction was transfected in cells plated on differential molecular tension probe‐engaged FN‐stripe micropatterns with or without FAK phosphorylation‐inhibiting PF573228 (Figure 5G–K vs L–P). Consistent with our persistent cell motion analysis (Figure 5A–F), cells plated on FN stripes engaged with tension probes bearing greater tolerance force were translocated further along the underlying micropatterns than cells plated on low force‐bearing probe‐engaged FN stripes; activated Cdc42 was more substantially localized at the front end of persistently migrating cells (Figure 5G–I; and Movie S7, Supporting Information). As expected, FAK phosphorylation‐inhibited cells did not display noticeable difference in the localization or translocation of activated Cdc42 in response to changes of subcellular molecular tension (Figure 5L–N; and Movie S7, Supporting Information). Plotting spatiotemporal changes where the highest Cdc42 activation was detected confirmed this notion, i.e., increasing integrin‐mediated molecular binding force maintained cell polarity at the front end of the cell (Figure 5J,K), but inhibiting FAK phosphorylation eliminated the integrin‐ligand binding force‐dependent cell polarization (Figure 5O,P).

Together, these results reveal that integrin subunit‐ligand binding strength regulates intracellular signal pathways via FAK phosphorylation, establishing cell polarity to maintain directional persistent cell migration.

### 2.6 Integrin‐Mediated Molecular Binding Force Recapitulates Global Mechanosensation of Substrate Compliance

Cell migration comprising continuous adhesion and de‐adhesion processes between subcellular focal adhesion clusters and ECM molecules is highly mechanosensitive to shift in substrate rigidity.^[^ ^44^, ^45^ ^]^ Therefore, we compared FAK phosphorylation‐dependent migration persistence in cells in contact with molecular tension probes and cells placed on substrates displaying varying elastic moduli to determine whether altering molecular binding force at a pico‐newton level specifically localized at the interface between a single integrin subunit and ECM ligand molecule could recapitulate collective cellular responses to global changes of the cell‐bearing physical environment.

**Figure 6:**
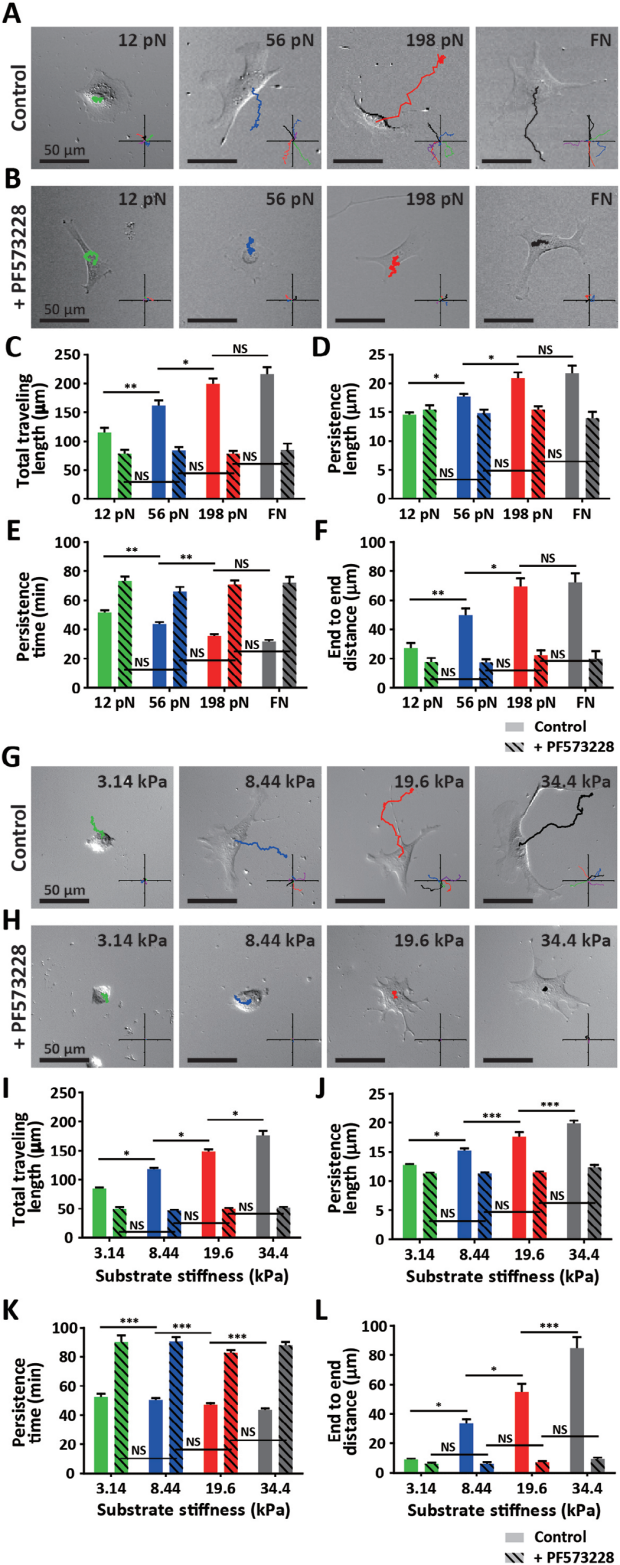
Recapitulation of substrate rigidity‐dependent differential persistent migration via integrin‐ligand binding force‐dependent modulation of persistent cell migration. A–F) Differential persistent migration in response to changes of integrin‐ligand binding strength. Traces of randomly migrating cells placed on molecular tension probe‐conjugated or bare FN substrates were time‐lapse monitored for 6 h with/without treatment with FAK phosphorylation inhibitor (PF573228) (A,B). *Insets* show representative trajectories for each condition, where increasing integrin‐ligand binding strength induced more persistent cell migration. Quantitative analysis of cell motility indicated that increasing integrin‐mediated molecular force enhanced total traveling length (C) and persistent motion (D,E), resulting in directional translocation (F), which was abrogated by inhibiting FAK phosphorylation (solid bars vs dashed bars, C‐F). G–L) Differential persistent cell migration in response to change of substrate rigidity. Traces of randomly migrating cells placed on FN‐coated polyacrylamide hydrogel substrates (elastic moduli ranges from 3.14 to 8.44 kPa, 19.66, and 34.4 kPa) were time‐lapse monitored for 6 h with/without treatment with FAK phosphorylation inhibitor (PF573228) (G,H). *Insets* display representative trajectories for each condition, where increasing substrate rigidity induced more persistent cell migration. Quantitative analysis of cell motility indicated that persistent cell migration induced by enhanced substrate rigidity was disrupted by inhibiting FAK phosphorylation (I–L). Persistent cell motion induced by global mechanosensation of substrate stiffness was recapitulated by local changes of integrin‐ligand binding force (A–F vs G–L). In panels C‐F and I‐L, > 20 cells were analyzed for each condition. Error bars indicate S.E.M.; statistical differences were calculated using one‐way ANOVA and Tukey's multiple comparison test. (NS: not significant; *: *p* < 0.01; **: *p* < 0.005; ***: *p* < 0.001).

We first monitored randomly migrating cells placed on differential molecular tension probe‐engaged FN substrates to characterize their persistence motion, followed by PF573228‐induced inhibition of FAK phosphorylation (**Figure** **6**A–F). Compared to cells confined on FN stripes that only allowed directional migration, unconfined cells exhibited the typical random migration, switching between persistent directional motion and non‐persistent meandering motion (Figures 5A vs 6A). Nevertheless, increasing integrin‐ECM binding strength enhanced cell motility, where trajectories of traveling cells placed on 198 pN‐probe‐anchored FN surfaces were primarily the same as that of cells on molecular tension probe‐absent FN surfaces (Figure 6A; and Movie S8, Supporting Information). However, inhibiting FAK phosphorylation disrupted cell migration and abrogated subcellular molecular binding force‐dependent alteration of cell migration, resulting in meandering cell migration resembling cells plated on FN surfaces (Figure 6B; and Movie S8, Supporting Information). Accordingly, while integrin‐ligand binding strength significantly increased the parameters determining cellular persistent movement and directional cell migration, such differences were not detected in PF573228‐treated cells (Figure 6C–F). These results confirmed that integrin‐mediated molecular binding force can regulate persistent directional cell migration via FAK phosphorylation.

Analysis of cell migration on the polyacrylamide (PA) hydrogel substrates coated with constant FN concentration, featuring differential elastic modulus ranging from 3.14 to 8.44, 19.6, and 34.4 kPa (Figure S8, Supporting Information), also revealed similar responses to changes of integrin‐ligand binding force (Figure 6G–L, and Movie S9, Supporting Information). PF573228‐induced inhibition of FAK phosphorylation also disrupted substrate stiffness‐dependent cell migration (Figure 6G,H), as confirmed by statistical analysis of total traveling distance (Figure 6I), persistence motion (Figure 6J,K), and directional cell migration (Figure 6L). Surprisingly, we noted that these parameter values were primarily in the same range as those obtained from changing the molecular tension probes (Figure 6C–F vs I–L). These results reveal that pico‐newton ranged manipulation of a single molecular‐specific binding force between integrin subunits and ECM ligands can control cell migration without altering the matrix elasticity that determines the global mechanical properties of extracellular microenvironments.

**Figure 7:**
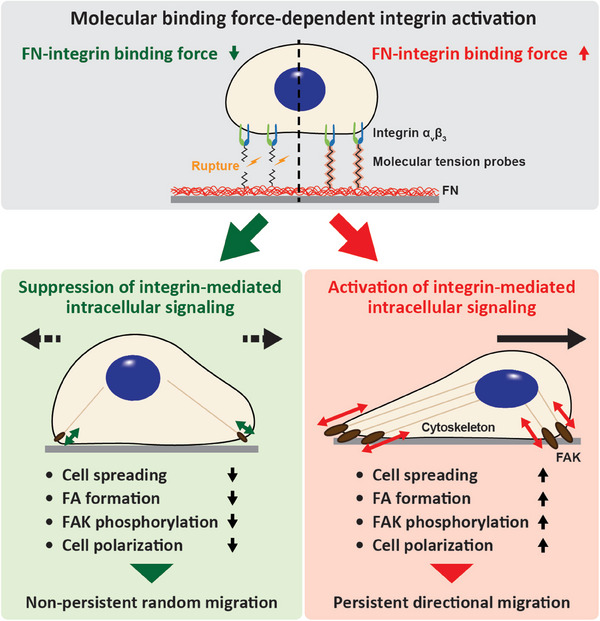
Schematic illustration depicting the integrin subunit‐ECM ligand binding force‐dependent intracellular signaling to determine directional cell migration. Modulation of integrin‐mediated intracellular signaling via precise control of molecular binding forces between integrin *α_v_β_3_* and FN regulates cell spreading, focal adhesion formation, FAK phosphorylation, and cell polarization, establishing persistent direction cell migration.

Together, our results demonstrate that attenuating the single molecular binding force at the interface of cell‐ECM contact, induced by breaking molecular links between integrin subunits and ECM ligand molecules, suppresses integrin‐mediated activation of intracellular signaling. Subsequently, this hinders FAK phosphorylation, focal adhesion formation, and cell polarization, resulting in non‐persistent random migration. In contrast, tight molecular linkage activated integrin‐mediated intracellular signaling to promote cell adhesion and persistent directional cell migration (**Figure** **7**).

## 3 Discussion

Our results demonstrated that the DNA‐based molecular tension probe‐assisted selective suppression of the binding force between an integrin subunit and ECM ligand molecule regulated cell adhesion by manipulating focal adhesion clustering that is tightly regulated by mechanical force balance between an integrin and substratum. Previous results indicated that cells preferentially adhere and migrate toward substrates with high rigidity^[^ ^7^ ^]^ or high ligand density^[^ ^,46^ ^]^ representing cellular mechanosensation of global surface properties. The molecular composition of the ECM and extracellular physical settings controls intracellular force transmission followed by force‐dependent intracellular signaling.^[^ ^47^, ^48^ ^]^ The force applied to the extracellular matrix is transmitted through conformational changes of integrin subunit, which activates focal adhesion proteins, determining the growth and maturation of clustered focal adhesions.^[^ ^49^, ^50^ ^]^ Collagen type I, a representative structural ECM molecules, enhances the activation of FAK, leading to the nuclear translocation of a transcription factor protein such as nuclear factor kappa‐light‐chain‐enhancer of activated B cells (NF‐κB)^[^ ^51^ ^]^ to induce the migration of adherent cells. However, our results revealed that subcellular manipulation of piconewton‐ranged binding force between an integrin subunit and a ligand molecule could also induce similar cellular responses. More importantly, we substantiate that such changes are regulated by dismantling a few molecular connections per cell, while most remain intact. Molecular tension probe‐anchored FNs occupying 1% of surface coverage revealed binding force‐dependent differential cell spreading (Figure 1). Molecular tension probes (12, 56, and 198 pN) that selectively regulate integrin activation can be considered by substituting global surface properties with different ligand densities^[^ ^52^, ^53^ ^]^ or integrin‐ECM interactions in substrate stiffness.^[^ ^7^ ^]^ Consistent with our results, a ligand density reduction induces integrin inactivation, inhibiting larger cell spreading and directional migration, forming smaller focal adhesions, and decreasing FAK phosphorylation.^[^ ^52^ ^]^ Furthermore, increasing ligand density more sensitively controls cell spreading, similar to increasing substrate stiffness.^[^ ^7^ ^]^ These results corroborate our findings that as the amount of ligand molecules increases by focal contact size, integrin‐mediated cell spreading responds more sensitively relative to the molecular binding force (Figure 2).

Transmission of intracellular force through a physical connection between a force transduction layer composed of focal adhesion complex proteins and an actin‐regulatory layer comprising actomyosin stress fibers induces conformational changes of various focal adhesion proteins during integrin‐mediated signal activation.^[^ ^54^ ^]^ Integrin progresses through several stages in the conformational change of integrin: bent‐closed, extended‐closed, and extended‐open. In the bent‐closed state, cytoplasmic proteins of integrin are in an inactivated form.^[^ ^55^ ^]^ Upon binding with an extracellular matrix ligand, the conformation of integrin changes to an extended state, activating of integrin and leading to the separation of integrin's cytoplasmic tails. This separation allows kindlin and talin to bind to the integrin β tail, which in turn induces actin binding and recruits the focal adhesion complex proteins such as FAK, vinculin, Src kinases, and the Arp 2/3 complex.^[^ ^56^ ^]^

Intracellular force generating by talin‐actin binding increases life time of catch bond of integrin, with stabilizing integrin‐mediated intracellular signaling, and induces clustering of surrounding integrins.^[^ ^56^ ^]^ Focal adhesion maturation occurs when hundreds of focal adhesion components form an interconnected network.^[^ ^57^ ^]^ In the early stage, talin, kindlin, and FAK bind to the extended‐open integrin and stimulate the recruitment of other focal adhesion proteins.^[^ ^58^ ^]^ Meanwhile, intermediate focal adhesion proteins, such as vinculin and paxillin, form a bridge between membrane‐mediated focal adhesion‐associated proteins and actin‐binding focal adhesion proteins. Later, zyxin and vasodilator‐stimulated phosphoprotein mediate F‐actin binding by the focal adhesion complex.^[^ ^59^ ^]^ Thus, the maturation process of focal adhesion induces the evolution of nascent focal adhesion to fibrillar adhesion.

On the other hand, while focal adhesions formed within 3D environments also undergo stages of formation, stability, and disassembly similar to those in 2D environment,^[^ ^60^ ^]^ the focal adhesions formed in 3D environment exhibit a relatively prolonged lifetime.^[^ ^61^, ^62^ ^]^ For instance, within 3D collagen hydrogels, zyxin reveals the extended adhering lifetime and the turnover rate,^[^ ^62^ ^]^ which was further intensified by increasing the stiffness of collagen fibrils composing the 3D collagen gels.

Moreover, vinculin and talin, primary components of a typical force transduction layer, require an average force of 2.5 and 10 pN during integrin activation.^[^ ^63^, ^64^ ^]^ FAK, which undergoes force‐induced conformation change with these proteins, triggers focal adhesion‐mediated intracellular signals for cell adhesion and migration via integrin‐ECM binding,^[^ ^65^ ^]^ because the N‐terminal FERM domain of FAK binds to the membrane and its C‐terminal FAT domain anchors to vinculin and actin fibers through paxillin. Conformation change of FAK is regulated by the tensile force acting on it.^[^ ^66^ ^]^ Thus, force generation in actin fibers pulls on the C‐terminal FAT domain of FAK, dissociating between the FERM and kinase domains by an ≈25 pN force.^[^ ^66^ ^]^ This intracellular force‐induced separation exposes the Tyr397 site, which binds to the Src protein and phosphorylates FAK.^[^ ^67^ ^]^ Our results revealed that the integrin‐mediated molecular binding force regulates FAK phosphorylation (Figure 4). In particular, attenuation of FAK phosphorylation in cells placed on 12 pN‐probe‐anchored FN surface was attributed to insufficient molecular binding force between integrin *α_v_β_3_* and FN ligand molecules that separate the kinase and FERM domains. Therefore, FAK phosphorylation increased as the integrin‐mediated molecular force was more dominant when switching from 56 to 198 pN probes, notably higher than the 40 pN threshold force that activates integrin‐mediated cell adhesion.^[^ ^5^ ^]^

In turn, the Src kinase binds to the phosphorylated Tyr397 via the SH2 domain, which activates phosphorylation at Tyr576 and Tyr577. Simultaneously, two PxxP motives in the C‐terminal of FAK bind to the SH3 domain of p130Cas and interact with the SH3 domain of GTPase, activating the GTPase regulator associated with FAK (GRAF).^[^ ^68^ ^]^ Thus through this force‐induced FAK phosphorylation, Cdc42 and Rac1 of the GTPase family are recruited and activated at the focal adhesion.^[^ ^69^ ^]^ Our results demonstrated that integrin‐mediated molecular binding force‐dependent FAK phosphorylation induced differential Cdc42 activation during cell migration (Figure 5G–P). Moreover, polarized cells with activated Cdc42 induced migration toward the front of the cell (Figure 5). Thus, this sequential molecular activation results in persistent directional migration, potentially guided by alteration of integrin‐mediated molecular force (Figure 6A–F) or substrate stiffness (Figure 6G–L).^[^ ^70^, ^71^ ^]^

Thus, FAK plays a central role in regulating proteins associated with mechanical force transmission, ranging from cell adhesion to migration, through N‐terminal domain binding to the cell membrane and C‐terminal domain binding force transduction layer. FAK, phosphorylated by the strong binding force between ECM and integrins, regulates the activation of associated focal adhesion molecules, ultimately converting mechanical force into intracellular signaling.^[^ ^72^ ^]^ FAK‐mediated signaling involves not only Src kinase binding and GTPase family recruitment but also controls force transmission through exosomes^[^ ^73^, ^74^ ^]^ and the remodeling of extracellular proteins at the intracellular level.^[^ ^75^, ^76^ ^]^ Because exosomes regulated by FAK could enhance cancer cell protrusion dynamics and invasion capabilities via microRNA^[^ ^73^ ^]^ FAK plays a key role in mechano‐regulation specifically controlling both external and internal signaling pathways.

Based on our findings, we conclude that selective suppression of integrin‐mediated molecular binding force regulates integrin activation at the cell membrane and activates intracellular signaling, resulting in cell adhesion, polarization, and migration (Figure 7). Furthermore, we confirmed that FAK phosphorylation‐dependent cell migration induced by integrin‐mediated molecular binding force could recapitulate cellular mechanosensation of substrates with differential rigidity.

## 4 Experimental Section

### Cell Culture and Drug Treatment

MEFs were cultured in Dulbecco's modified Eagle's medium (DMEM, Corning, NY, USA), supplemented with 10% fetal bovine serum (FBS; Merck, NJ, USA), and 1% penicillin/streptomycin solution (Gibco, NY, USA). Cells were maintained in a 5% CO_2_ humidified incubator at 37 °C. MEFs were cultured on a 50 µg ml^−1^ FN‐coated 35 mm glass bottom dish (101 350, SPL, Korea) anchored with differential strengths of molecular tension probes. Cells were seeded at a 1 × 104cells ml^−1^ density on a glass bottom dish coated with integrin‐mediated molecular tension probe‐engaging FN. To degrade DNA‐based molecular tension probes, 20 units ml^−1^ DNase 1 (89 836, Thermo Fisher, USA) was added to the cell culture media and incubated for 1 h before cell fixation. To inhibit FAK phosphorylation at Tyr397, PF573228 (PZ0117, Sigma‐Aldrich, USA) was used at a final 10 µm concentration in DMEM.

### Transient Transfection

MEFs were transfected using Lipofectamine 3000 reagent (Invitrogen, MA, USA) in Opti‐MEM reduced serum media (Gibco, MA, USA) following the manufacturer's instructions. Cells were transfected with the FRET‐based cytosolic FAK biosensor plasmid (78 300, Addgene, MA, USA) or Raichu‐CDC42 that was provided by Professor Won Do Heo (Korea Advanced Institute of Science and Technology, Korea), with permission from Professor Michiyuki Matsuda (Kyoto University, Japan). After 48 h incubation with plasmids, cells were transplanted onto an integrin‐mediated molecular tension probe surface.

### Preparation of Integrin‐Mediated Molecular Tension Probes

Cyclopeptide RGDfK (biotin‐PEG‐PEG) (MBS405617, MyBioSource, USA) was prepared as a PEG‐based molecular tension probe. The tolerance force of this molecular tension probe was ascertained based on the neutravidin and biotin interaction. The probe was used at a final 10 µm concentration. For rupture trace experiments, a fluorescent dye was conjugated to a molecular force sensor.^[^ ^77^ ^]^ Three distinct single‐stranded DNAs were designed to formulate the DNA‐based molecular tension probes across integrin (top single‐strand DNA and two bottom single‐strand DNAs conjugated with biotin). The biotin‐tagged site determined the tolerance strength of the DNA‐based molecular tension probes, where it anchors to the surface via biotin and neutravidin interactions. Sequences and modifications for the top single‐stranded DNA and two bottom single‐strand DNA (Macrogen, Korea) are as follows:

Top single‐strand DNA: 5‐/5Thiol C6 S‐S/CAC AGC ACG GAG GCA CGA CAC‐3
Bottom single‐strand DNA (1): 5‐/5′Biotin/GTG TCG TGC CTC CGT GCT GTG‐3
Bottom single‐strand DNA (2): 5‐GTG TCG TGC CTC CGT GCT GTG‐/3′Biotin/3

DNA‐based molecular tension probes were synthesized as previously described.^[^ ^5^, ^78^ ^]^ For tethering integrin *α_v_β_3_* targeting peptide, cyclo peptide RGDfK‐NH2 was conjugated to the top single‐stranded DNA via the hetero‐bifunctional cross‐linker Sulfo‐SMCC (22 322, Thermo Scientific, MA, USA). More specifically, 1 mm of the top single‐stranded DNA in phosphate‐buffered saline (PBS) was dissolved in a mixed solution (50 mm TCEP + 50 mm EDTA, pH 7.2–7.4) for 30 min at room temperature (RT). Concurrently, another solution was prepared by mixing 50 µl of 10 mm RGDfK‐NH2 in PBS with 10 µl of 23 mm sulfo‐SMCC for 20 min at RT. After mixing both prepared solutions, they were allowed to react for 1 h at RT. Next, the RGDfK peptide‐conjugated top single‐stranded DNA was assembled with two biotin‐tagged bottom single‐strand DNAs in PBS. The final concentration of the DNA‐based molecular tension probes was 10 µm. The DNA‐based molecular tension probe coupled to the bottom single‐stranded DNA with the biotin 5′ end had a 12 pN tolerance force, while another DNA‐based molecular tension probe exhibited a 56 pN tolerance force. For rupture trace experiments necessitating visualization, the top single‐stranded DNA with Cy5 at the 3′ end was used to anneal and form the DNA‐based molecular tension probes.

### Immobilization of Molecular Tension Probes

A glass bottom dish was pre‐coated with 50 µg ml^−1^ FN (F0895, Sigma‐Aldrich, MO, USA) for 8 h at 4 °C to immobilize the integrin‐mediated molecular tension probes. After washing away unbound FN with PBS three times, the glass bottom dish was treated with varying concentration of biotin‐conjugated FN antibody (0, 0.2, 1, 4, 10, and 100%) for 1 h at RT. Then, the glass bottom dish was incubated with 200 µg ml^−1^ neutravidin (31 000, Thermo Fisher Scientific, MA, USA) for 30 min at RT. Finally, the surface was coated with 10 µm of integrin‐mediated molecular tension probes for 30 min at 4 °C, washed three times with PBS, and cells were immediately plated on integrin‐mediated molecular tension probe‐anchored surfaces.

### Fibronectin Micropatterning

A SU‐8‐based master mold for the microsized island (1, 3, and 5 µm) and the 20 µm line micropattern were fabricated using soft lithography to prepare FN‐micropatterned substrates via microcontact printing. For the microsized island mold, a silicon wafer was spin‐coated with SU8‐2 photoresist (Microchem, MA, USA) at 3500 rpm for 40 s, achieving a 1 µm thickness.^[^ ^31^ ^]^ For the 20 µm line micropattern mold, a wafer was coated with SU8‐10 photoresist (Microchem, MA, USA) at 3000 rpm for 30 s, forming a 10 µm thickness. Both wafers underwent a two‐step soft‐bake: at 65 °C for 1 and 2 min, then at 95 °C for 1 and 5 min. After UV irradiation exposure (80 mJ cm^−2^) using custom‐designed photomasks, they were hard‐baked at 65 °C for 1 min and at 95 °C for 1 min and 2 min, respectively. Following etching with the SU8 developer (Kayaku, MA, USA), a polydimethylsiloxane (PDMS, Sylgard 184, Dow Corning, MI, USA) prepolymer (10:1 ratio of elastomer to curing agent) mixture was poured into each master mold and baked at 70 °C for 10 h in an oven. Cured PDMS blocks were removed from the master molds, and PDMS stamps were cut from these blocks. After sterilization in ethanol, the PDMS stamps were incubated with 50 µg ml^−1^ rhodamine‐fibronectin (Cytoskeleton, CO, USA) for 30 min. The PDMS stamps were dried entirely to remove any remaining solution and were placed on oxygen plasma‐treated (600 mTorr, 60 W for 3 min) flux confocal dishes (211 350, SPL, Korea) for 1 min. Finally, the plates were passivated with 0.1 mg ml^−1^ Poly(L‐Lysine)(20)‐grafted[3.5]‐PEG(2) (SuSoS AG, Switzerland) in 10 mm HEPES (pH 7.4) for 60 min to block adherent cells on non‐coated surfaces.

### Immunostaining and Immunofluorescence Microscopy

Cells were fixed with 4% paraformaldehyde (Electron Microscopy Sciences, USA) for 10 min at 4 °C, then washed with PBS three times. Next, cells were permeabilized using the 0.01% Triton‐X for 10 min at RT. After washing with a washing buffer (PBS containing 0.1% FBS), cells were treated with 10% FBS in PBS for 30 min to block non‐specific binding. To visualize and analyze focal adhesion formation and FAK expression, cells were incubated for 1 h with monoclonal anti‐vinculin antibody (100:1, MA5‐15588, Thermo Fisher Scientific, MA, USA) and monoclonal anti‐FAK antibody (100:1, V4505, Sigma‐Aldrich, MO, USA), respectively. Then, cells were stained for 1 h with DAPI (0.5 µg ml^−1^ in PBS), Alexa Fluor 488 phalloidin (400:1, A12379, Invitrogen, MA, USA), and Alexa Fluor 594 goat‐anti‐mouse (500:1, Benthyl, AL, USA). For FAK phosphorylation detection, blocking buffer treated‐cells were stained with DAPI, Alexa Fluor 488 phalloidin, and anti‐phosphorylated FAK in Tyr397 antibody (200:1, bs‐3159R‐A594, Bioss, MA, USA) for 1 h. Following each treatment, cells were washed with PBS three times. Immunofluorescence images were captured using confocal laser scanning microscope (Nikon A1R, Nikon, Japan) or fluorescence microscope (Nikon Ti2‐E, Nikon, Japan) and then processed with NIS‐elements software (Nikon, Japan).

### Cell Morphometry, Protein Content, and Cell Cycle Measurements

Stained cells were imaged using a multimode imaging reader (Cytation 3, BioTek, VT, USA) with DAPI, GFP, and Texas‐red filter cubes. A 10× flat lens was used to obtain immunofluorescence images. Captured images were analyzed using custom MATLAB codes and NIS‐elements software to measure individual cell morphology and immunofluorescence intensity (Figures 1 and 4). GFP channel images facilitated cell region identification; DAPI channel images were used to segment nuclei, with the same threshold applied to all images. Fluorescence intensity, which indicates immunostained protein contents, was quantified based on the segmented cell area in Texas‐red channel images. To analyze the cell cycle, fluorescence intensity of DAPI in each segmented nucleus was quantified and their fraction was calculated.

### Live Cell Imaging

Cells were plated on a glass bottom dish and incubated in a microscope stage top chamber (Okolab, Napoli, Italy) at 37 °C with 5% CO_2_ for live cell imaging. All live images were captured with confocal laser scanning microscope and fluorescence microscope. Time‐lapse images were obtained with a Plan Apo VC 20× and 60× oil lens (Nikon, Japan). Total and specific time intervals for these live image sequences are detailed in the respective figure captions.

### Analysis of Förster Resonance Energy Transfer (FRET) Biosensor Signals

MEFs were transfected with a cytosolic FAK FRET sensor (Addgene, MA, USA)^[^ ^40^ ^]^ and Raichu‐Cdc42 plasmids^[^ ^79^ ^]^ using a Lipofectamine 3000 reagent to monitor FAK and Cdc42 activation. Cytosolic FAK plasmids incorporated ECFP and YPET fluorescent proteins, while Raichu‐Cdc42 plasmids contained CFP and YFP proteins. For FRET imaging, donor proteins (ECFP and CFP) were excited using a 405 nm laser, while acceptor proteins (YPET and YFP) were excited using a 488 nm laser with a confocal microscope. These FRET images were analyzed using the “PixFRET” plug‐in in Image J Fiji^[^ ^80^ ^]^ and visualized using the conventional fire code in ImageJ. The FRET ratio was determined by dividing the intensity of the acceptor proteins by that of donor proteins. For analysis of FAK FRET signal, relative fluorescence intensity by calculating the ECFP and YPET in the cytoplasm to the nucleus. For the Raichu‐Cdc42 FRET sensor, the highest intensity point was identified using a custom MATLAB code.

### Cell Migration Analysis

Live cell images were captured over time using a confocal microscope. These images were collected using the NIS elements program to analyze cell motility. Cell nuclei in each image were traced to obtain centroid X‐ and Y‐positions. The total traveling length was calculated as the sum of distances between consecutive nucleus centroids over time. Persistent length was defined as a straight movement of more than 10 µm without changing a direction angle over 70°. Persistence time was defined as the average duration for each persistent length. End‐to‐end distance was calculated as the distance between initial and final X and Y positions of the cell.

### Preparation of Polyacrylamide Hydrogel Substrates

For preparing polyacrylamide (PA) gel substrates, glass bottom dishes were sequentially treated with 3‐Aminopropyltrimethoxysilane (281 778, APTMS, Sigma‐Aldrich, MO, USA) for 10 min and 0.5% glutaraldehyde (G6257, Sigma‐Aldrich, MO, USA) for 30 min. The PA‐gel premixture was prepared using acrylamide (A8887, Sigma‐Aldrich, MO, USA) and N,N'‐methylene bisacrylamide (M7279, Sigma‐Aldrich, MO, USA) in a 1:1 ratio. Then, 0.05% ammonium persulfate (A3678, Sigma‐Aldrich, MO, USA) and 0.15% N,N,N´,N´‐tetramethylethylenediamine (T9281, Invitrogen, MA, USA) were added into the mixture. PA hydrogel stiffness was determined by concentrations of acrylamide and bisacrylamide.^[^ ^81^ ^]^ The mixed PA solution was incubated in pre‐treated glass bottom dishes and covered with a dichlorodimethylsilane (Sigma‐Aldrich, MO, USA)‐treated glass coverslip to flatten the gel. After polymerization, the coverslip was removed from the PA gel. For functionalization of the PA hydrogel surface, 50 mm sulfosuccinimidyl‐6‐(4′‐azido‐2′‐nitrophenylamino) hexanoate (22 589, sulfo‐SANPAH, Thermo Fisher Scientific, MA, USA), a heterobifunctional cross‐linker, was supplemented on the PA hydrogel and exposed to UV light for 5 min. After washing in 200 mm HEPES, the PA hydrogel was coated with 50 µg ml^−1^ FN and left overnight. Experiments were performed after washing with PBS before use.

### Neutravidin‐Biotin Binding Force Measurement

AFM cantilevers (PNP‐TR‐20, Nanoworld, Switzerland) were used to measure neutravidin‐biotin binding forces. Prior to measurements, cantilevers were functionalized with PEG‐biotin (QBD10200, Sigma‐Aldrich, MO, USA)^[^ ^82^ ^]^ and treated using oxygen plasma at 600 mTorr and 100 W for 10 min. For forming amino groups, cantilevers were immersed in a 5% APTMS and absolute ethanol solution for 12 h. After rinsing with toluene, cantilevers were incubated in 1 mg ml^−1^ PEG‐biotin in chloroform with 0.5% trimethylamine catalysts for 2 h. Neutravidin‐biotin binding forces were measured using an AFM (NX‐10, Park systems) under 0.1 µm ^−1^s in a PBS more than 50 points.

### Tunel Assay

To perform the terminal transferase dUTP nick‐end labeling (Tunel) assay, the In Situ Cell Death Detection Kit (11 684 795 910, Roche, Germany) was used, where cellular apoptosis was determined by quantifying the labeled deoxyuridine triphosphate (dUTP) at DNA break sites. The percentage of the number of Tunel signal‐positive cells relative to the total number of cell nuclei was measured based on fluorescence images. The Tunel assay was performed on the control cells cultured with DMSO and 10 µm of PF573228‐treated cells, followed by fixation and permeabilization, and then co‐stained with DAPI. The concentration of Tunel assay was determined according to the protocol provided by a manufacturer.

### Statistical Analysis

All statistical analyses, including mean values and error bars, were conducted using GraphPad Prism 5 (GraphPad Software, USA). Sample numbers are indicated in the figure captions. Significance was determined using a one‐way ANOVA followed by Tukey's multiple comparison test. Error bars depict the standard error of the mean (S.E.M.), and all experiments were performed at least three times.

## Conflict of Interest

The authors declare no conflict of interest.

## Author Contributions

S.B.H. and G.L. contributed equally to this work. S.H. and G.L. designed, performed, and analyzed all experiments unless otherwise specified and co‐wrote the manuscript; D.S.K. and H.K. co‐wrote the manuscript; J.K. designed the image analysis method; I.K. and H.K. provided a conceptual design of the experimental settings and interpreted data. D.K. supervised the project, designed the experiments, and wrote the manuscript.

## Supporting information

Supporting Information

Supplemental Movie 1

Supplemental Movie 2

Supplemental Movie 3

Supplemental Movie 4

Supplemental Movie 5

Supplemental Movie 6

Supplemental Movie 7

Supplemental Movie 8

Supplemental Movie 9

## Data Availability

The data that support the findings of this study are available from the corresponding author upon reasonable request.
