# Supplementary material for: Selective Suppression of Integrin‐Ligand Binding by Single Molecular Tension Probes Mediates Directional Cell Migration
Source: Adv Sci (Weinh). 2024 Feb 4;11(14):2306497. doi: 10.1002/advs.202306497 (PMC11005741; doi:10.1002/advs.202306497)
Supplement: Supplementary file 1 — Supporting Information [file ADVS-11-2306497-s006.pdf]

ADVANCED SCIENCE
Open Access

Supporting Information

for *Adv. Sci.*, DOI 10.1002/advs.202306497

Selective Suppression of Integrin-Ligand Binding by Single Molecular Tension Probes Mediates Directional Cell Migration

*Seong-Beom Han, Geonhui Lee, Daesan Kim, Jeong-Ki Kim, In-San Kim, Hae-Won Kim and Dong-Hwee Kim**

Selective Suppression of Integrin-Ligand Binding by Single Molecular Tension Probes Mediates Directional Cell Migration

Seong-Beom Han[1,#], Geonhui Lee[1,#], Daesan Kim[1], Jeong-Ki Kim[1], In-San Kim[1,2], Hae-Won Kim[3,4], and Dong-Hwee Kim[1,2,5,*]

[1] KU-KIST Graduate School of Converging Science and Technology, Korea University, Seoul 02841, Republic of Korea

[2] Biomedical Research Center, Korea Institute of Science and Technology, Seoul 02792, Republic of Korea

[3] Institute of Tissue Regeneration Engineering (ITREN), Dankook University, Cheonan 31116, Republic of Korea

[4] Department of Biomaterials Science in College of Dentistry & Department of Nanobiomedical Science in Graduate School, Dankook University, Cheonan 31116, Republic of Korea

[5] Department of Integrative Energy Engineering, College of Engineering, Korea University, Seoul, 02841, Republic of Korea

***** The supplementary information includes following data *****

Supplementary Figure 1

Supplementary Figure 2

Supplementary Figure 3

Supplementary Figure 4

Supplementary Figure 5

Supplementary Figure 6

Supplementary Figure 7

Supplementary Figure 8

Supplementary Movie 1 (still image and legend)

Supplementary Movie 2 (still image and legend)

Supplementary Movie 3 (still image and legend)

Supplementary Movie 4 (still image and legend)

Supplementary Movie 5 (still image and legend)

Supplementary Movie 6 (still image and legend)

Supplementary Movie 7 (still image and legend)

Supplementary Movie 8 (still image and legend)

Supplementary Movie 9 (still image and legend)

Supplementary Figure 1

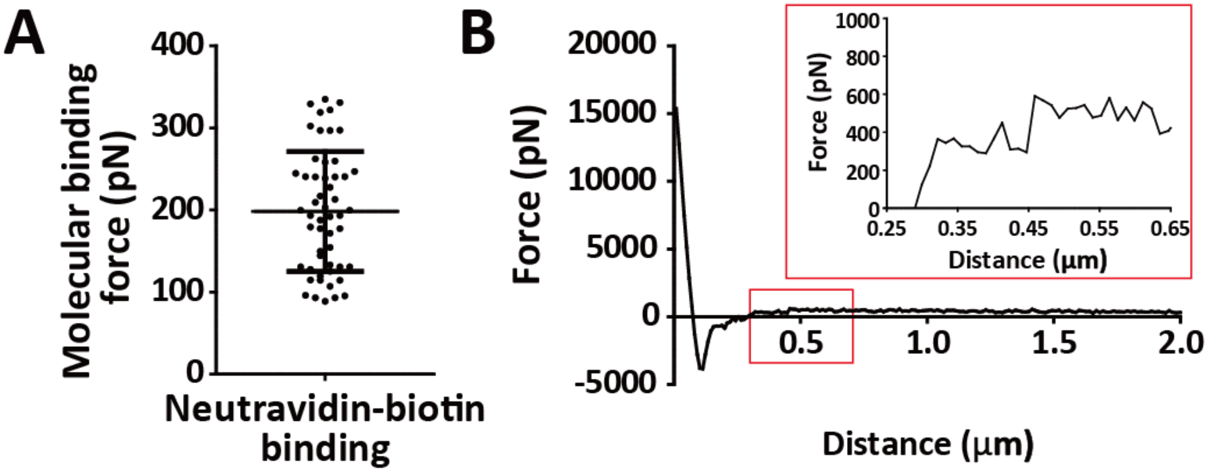

Supplementary Figure 1. Force-displacement curves of the neutravidin–biotin dissociation. **A**. Molecular binding forces between neutravidin and biotin were measured at a 0.1 μm/s AFM loading rate. > 50 points were measured. **B**. A representative force-displacement curve of neutravidin-biotin binding indicates the force change from molecular rupture between neutravidin and biotin (red box).

Supplementary Figure 2

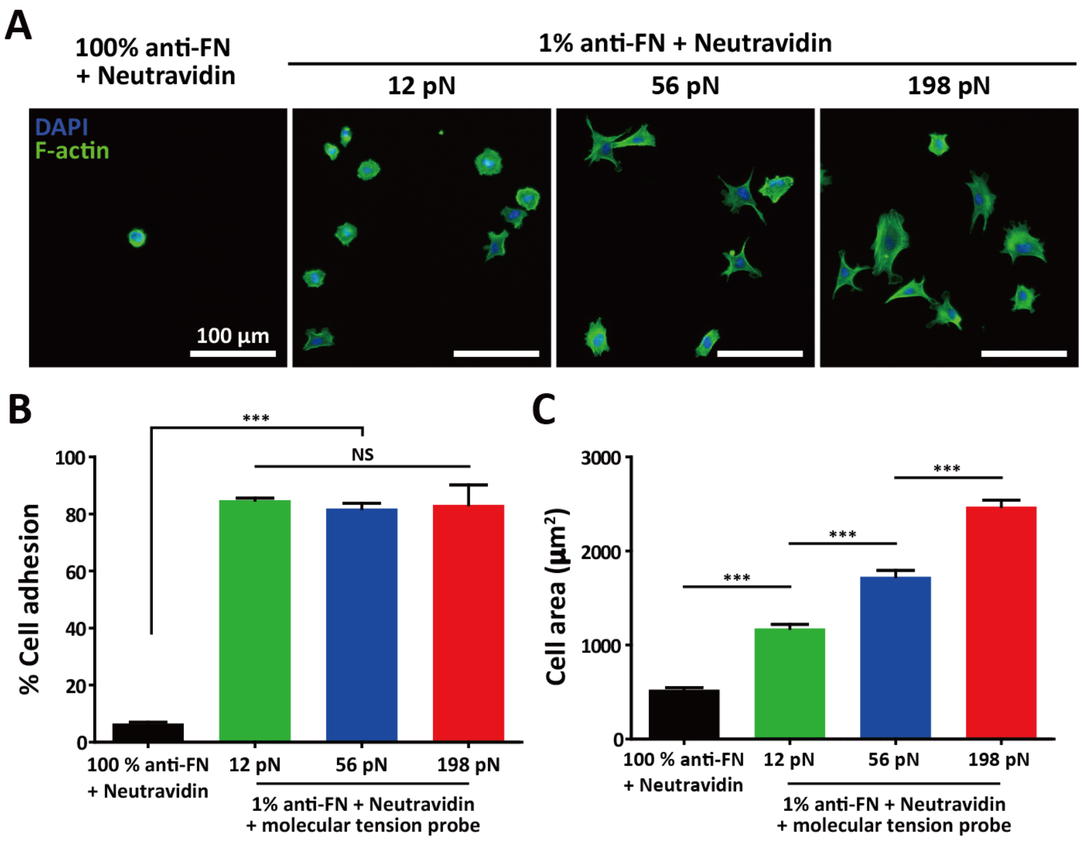

Supplementary Figure 2. Prevention of cell adhesion and spreading by coating the anti-FN antibody. A. Representative confocal images depicting cell adhesion and spreading on the FN-coated substrates that covered by differential content of FN-antibodies and molecular tension probes. FN-coated substrates were either covered by 100 % of biotin-conjugated FN antibody-neutravidin assemblies or 1 % of biotin-conjugated FN antibody anchored with molecular tension probes (12 pN, 56 pN, and 198 pN). MEFs placed on the surface were immuno-stained to visualize the nucleus (DAPI, blue) and F-actin (green). While cells were rarely attached to the 100% biotin-coupled FN antibody-neutravidin-coated surfaces, 1% biotin-coupled FN antibody-neutravidin-molecular tension probe-coated surfaces accommodated cell adhesion and modulated cell spreading area by molecular tension probes. Scale bars, 100 μm. **B-C.** Statistical analysis of the fractional cell adhesion and cell spreading, quantified by the number of adhered cells to the number of seeded cells and averaged spreading area of adhered cells, respectively. In panels A-C, > 300 cells were analyzed for each condition. Error bars indicate S.E.M.; statistical differences were assessed by one-way ANOVA and Tukey's multiple comparison test. (NS: not significant; ***: $p < 0.001$)

Supplementary Figure 3

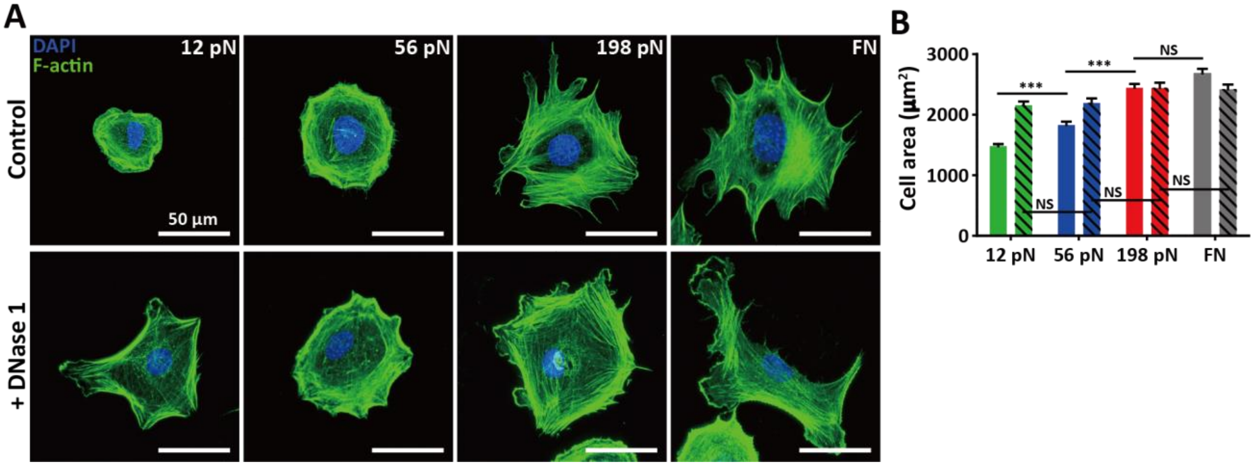

Supplementary Figure 3. Modulation of cell spreading in response to degradation of DNA-based molecular tension probes.

A. Degradation of molecular tension probe by treatment of DNase 1 abrogated the molecular tension probe-specific alteration of cell spreading. MEFs placed on FN-coated substrates with molecular tension probes (12, 56, and 198 pN) were immuno-stained with nucleus (DAPI, blue) and F-actin (green) before and after treating with dsDNA degrading DNase 1. **B**. Statistical analysis of cell spreading area changed by differential molecular tension probes following DNase 1 treatment. While the cell spreading area remained unchanged in 198 pN-probe-engaged surfaces, DNase 1 treatment abrogated the molecular tension-dependent differential changes of cell spreading. In panel B, > 250 cells were analyzed for each condition. Error bars indicate S.E.M.; statistical differences were assessed by one-way ANOVA and Tukey's multiple comparison test. (NS: not significant; ***: $p < 0.001$)

Supplementary Figure 4

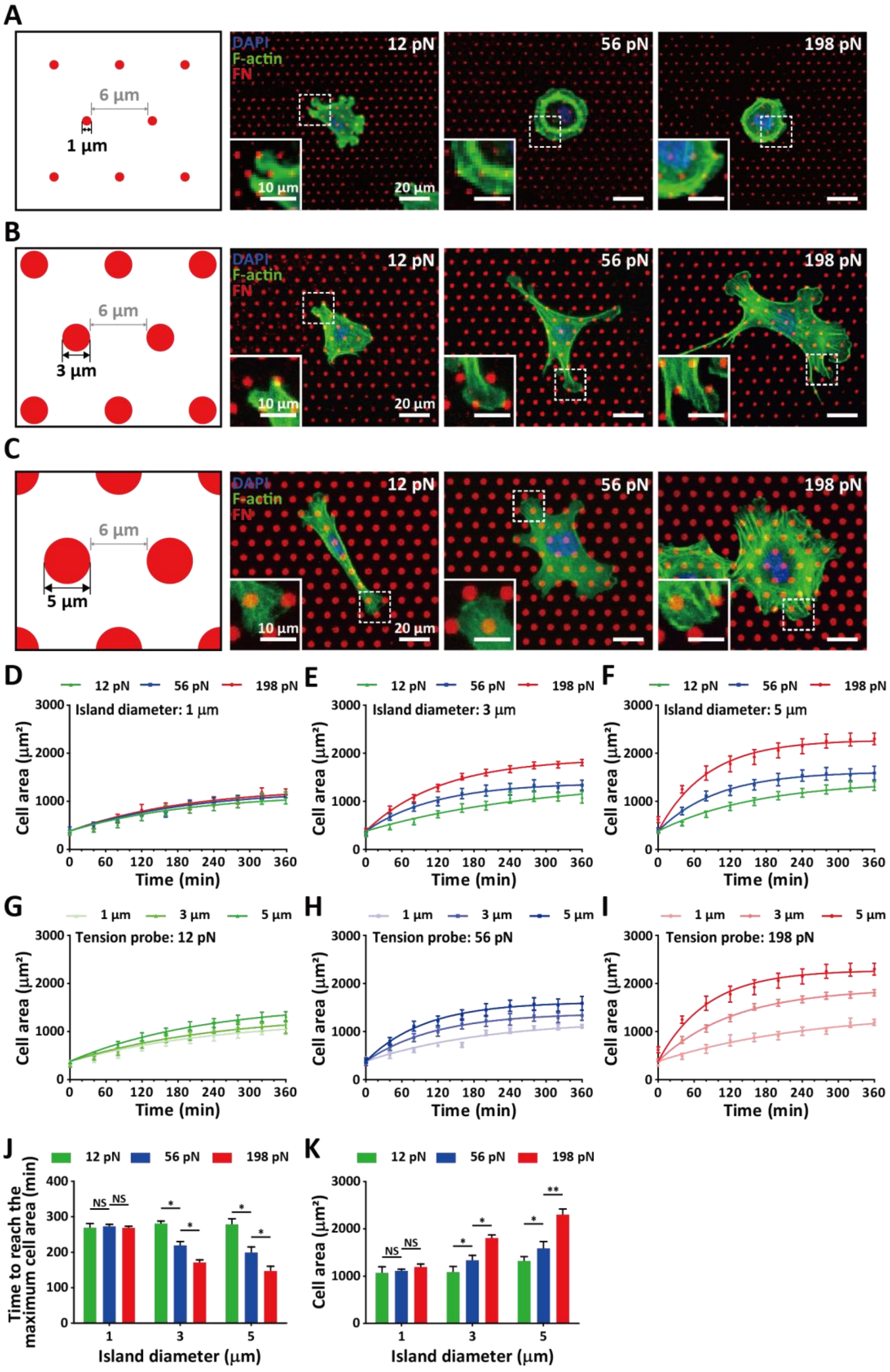

Supplementary Figure 4. Modulation of cell spreading in response to differential surface coverage with constant gap distance between molecular tension probe-anchored focal contacts. A-C. Representative confocal images depicting differential cell spreading in response to integrin $\alpha_v\beta_3$-mediated molecular binding forces in a confined cell-ECM contact maintaining a fixed gap distance. MEFs placed on size-controlled circular islands with 6 μm of gap distance between neighboring FN islands were immuno-stained to visualize the nucleus (DAPI, blue) and F-actin (green), where diameters of FN circular islands were 1 μm, 3 μm, and 5 μm, respectively. **D-I.** Time-dependent cell spreading dynamics in response to molecular binding strength and cell-ECM contact size. Cell spreading area of MEFs was captured every 20 min for 6 h. Cells were placed on the substrates with varying diameter of FN-circular islands (1 μm (D), 3 μm (E), and 5 μm (F)) with a fixed gap distance (6 μm), which were conjugated with molecular tension probes gauging 12 pN (G), 56 pN (H), and 198 pN (I), respectively. **J-K.** Quantification of differential cell spreading dynamics in response to partial changes of integrin $\alpha_v\beta_3$-mediated molecular binding force within the controlled cell-ECM contact with the identical gap distance. Not that the acceleration of cell adhesion and spreading in response to integrin $\alpha_v\beta_3$-mediated molecular binding force was more significant on the cells placed on the enlarged focal contact. In panels D-K, > 20 cells were analyzed for each condition. Error bars indicate S.E.M. Statistical differences were calculated using one-way ANOVA and Tukey's multiple comparison test. (NS: not significant; *: $p < 0.01$; **: $p < 0.005$).

Supplementary Figure 5

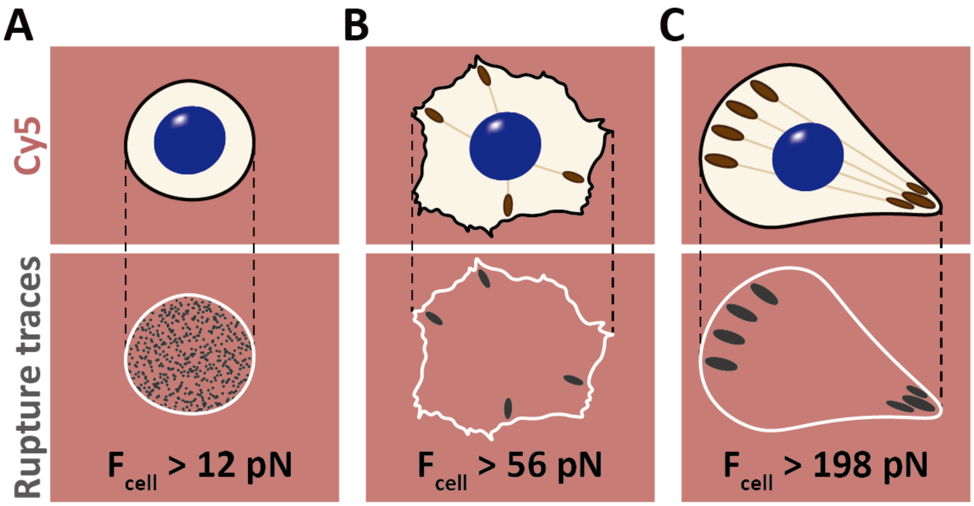

Supplementary Figure 5. Schematic presentation of molecular tension-dependent differential rupture traces of FN-coated substrates. Because molecular tension probes were ruptured when the integrin-mediated molecular force was larger than the threshold force of molecular tension probes, subcellular force surpassing the molecular binding force between integrin $\alpha_v\beta_3$ and FN-coated substrates ruptures molecular tension probes, leaving the dark spots. This result indicated that fluorescence molecule Cy5 bound to the upper ssDNA in 12 pN-probes (A) and 56 pN-probes (B) or PEG measuring the 198 pN-probes (C) were dissociated from the molecular tension probe-engaged FN-coated surfaces. Cells weakly adhere to the 12 pN-probes-engaged FN-coated surfaces formed the scattered rupture traces in whole cell area, whereas cells on the 56 pN-probes and 198 pN-probes-engaged FN-coated surfaces left concentrated rupture traces along the focal adhesions located in the periphery of the adherent cells.

Supplementary Figure 6

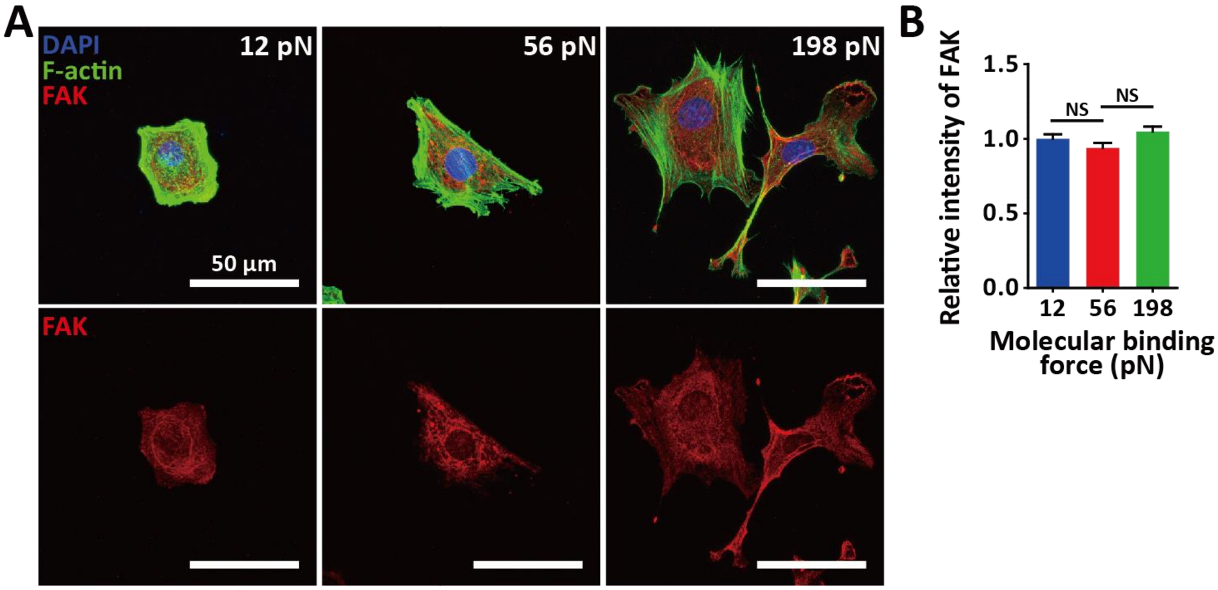

Supplementary Figure 6. Differential FAK expressions in response to integrin-ligand binding force.

A. Representative fluorescence images display the differential integrin-ligand binding force-dependent FAK expressions. MEFs placed on FN-coated substrates conjugated with differential molecular tension probes were immuno-stained for FAK (red), nuclear DNA (DAPI, blue), and F-actin (green) after 3 h of cell plating. **B**. Quantitative analysis indicated that total fluorescence intensity of FAK did not respond to changes of integrin-ligand binding strength. In panel B, > 300 cells were analyzed for each condition. Error bars indicate S.E.M.; statistical differences were assessed by one-way ANOVA and Tukey's multiple comparison test. (NS: not significant)

Supplementary Figure 7

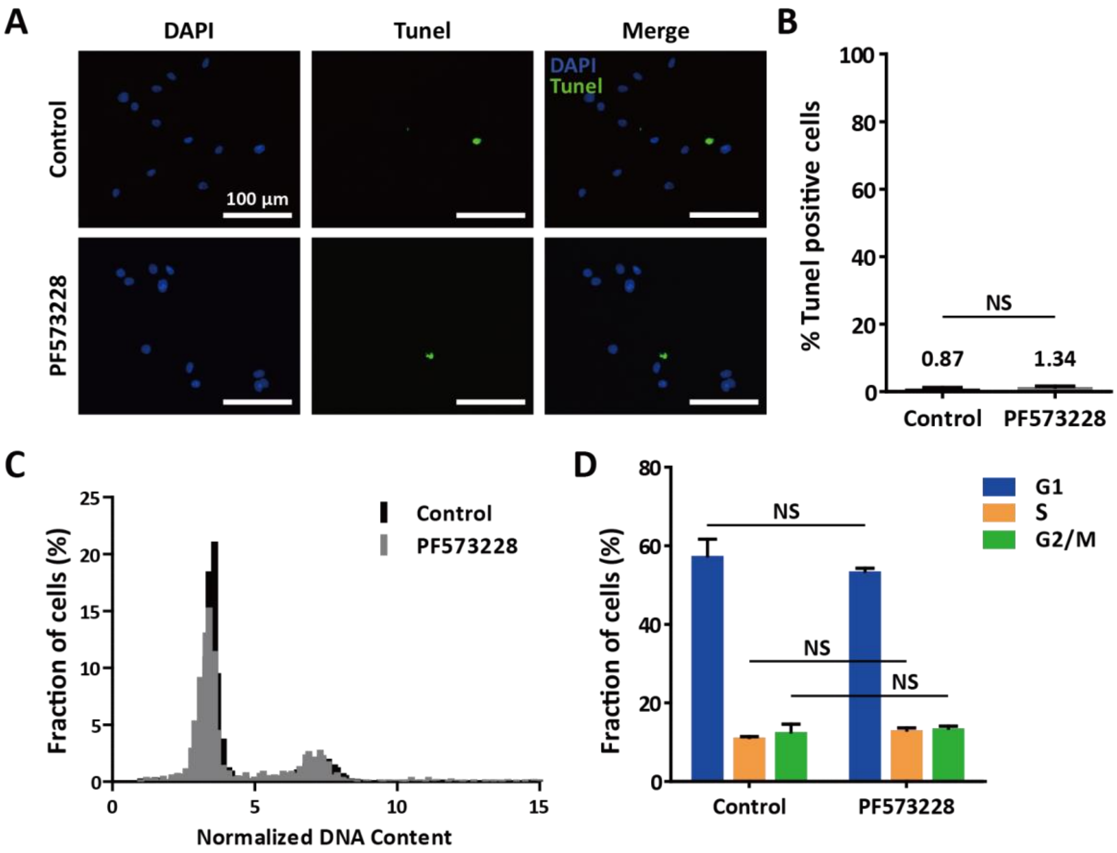

Supplementary Figure 7. Evaluating the effect of pharmaceutical inhibition of FAK activity by PF573228 on the apoptosis and cell cycle. A-B. Assessment of cell apoptosis in control and PF573228-treated cells. Representative fluorescence images of Tunel assay of control (DMSO) and PF573228-treated conditions. MEFs were marked by nuclear DAPI (blue) and apoptosis signal was shown green (A). The quantification of cell apoptosis signal was performed by quantification of Tunel positive cells relative to the total cell count (B). **C-D.** DNA content-based analysis of cell cycle in control and PF573228-treated cells. Cell count distribution depicting the differential DNA content was plotted in control (black) and PF573228-treated cells (gray) (C). No changes of cell cycle distribution were detected between control and PF573228 treatment (D). In panels A-D, > 300 cells were analyzed for each condition. Error bars indicate S.E.M. Statistical differences were assessed by either t-test (B) or one-way ANOVA followed by Tukey's multiple comparison test (D). (NS: not significant).

Supplementary Figure 8

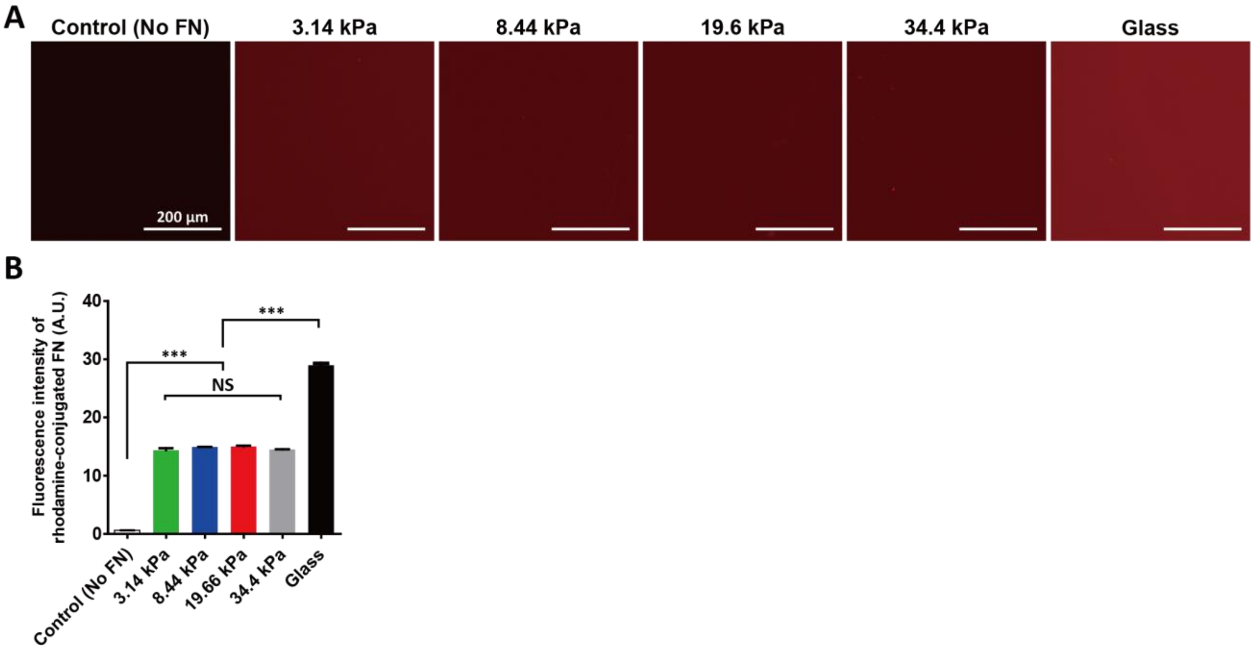

Supplementary Figure 8. Controlled presentation of FN on the surface of PA hydrogels. A. Representative fluorescence images of control bare glass (No FN) and rhodamine-conjugated FN-coated PA hydrogels with varying elastic moduli (3.14, 8.44, 19.6, and 34.4 kPa) and glass surface. Scale bars, 200 µm. **B.** Quantification of fluorescence intensity of rhodamine-conjugated FN. Error bars indicate S.E.M.; statistical differences were assessed by one-way ANOVA and Tukey's multiple comparison test. (NS: not significant; ***: $p < 0.001$)

Supplementary Movie 1

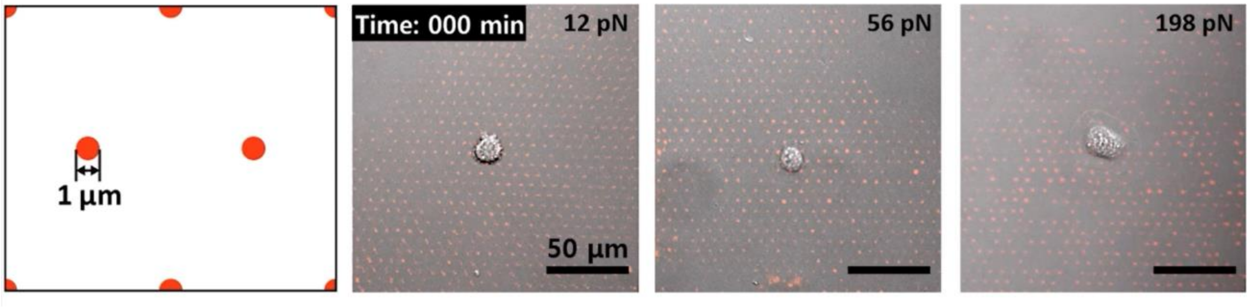

Supplementary Movie 1. Cell spreading dynamics on molecular tension probe-coated 1 µm FN-island surfaces.
Cell spreading dynamics on differential molecular tension probe-coated 1 µm FN-island surfaces were time-lapse monitored every 10 min for 6 h on 12 pN tension probe-coated surfaces (left), 56 pN-coated surfaces (middle), and 198 pN-coated surfaces (right).

Supplementary Movie 2

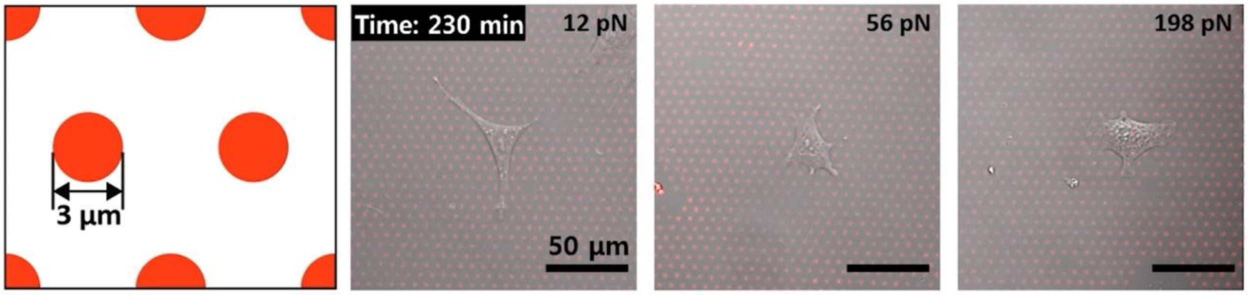

Supplementary Movie 2. Cell spreading dynamics on molecular tension probe-coated 3 µm FN-island surfaces.

Cell spreading dynamics on differential molecular tension probe-coated 3 µm FN-island surfaces were time-lapse monitored every 10 min for 6 h on 12 pN tension probe-coated surfaces (left), 56 pN-coated surfaces (middle), and 198 pN-coated surfaces (right).

Supplementary Movie 3

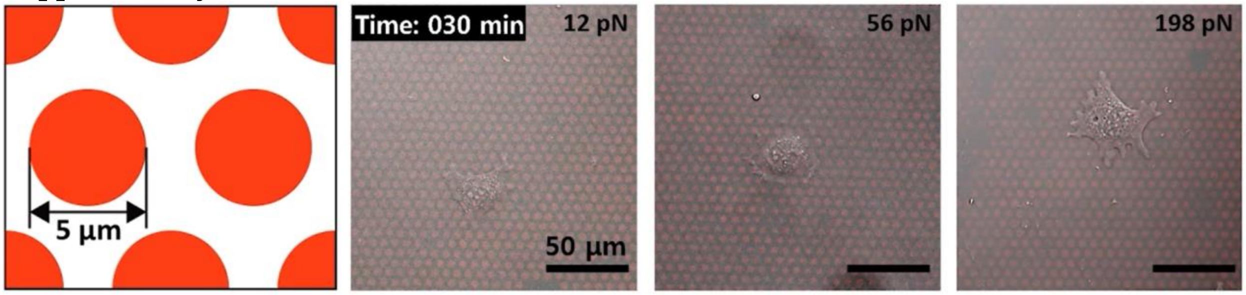

Supplementary Movie 3. Cell spreading dynamics on molecular tension probe-coated 5 μm FN-island surfaces.

Cell spreading dynamics on differential molecular tension probe-coated 5 μm FN-island surfaces were time-lapse monitored every 10 min for 6 h on the 12 pN tension probe-coated surfaces (left), 56 pN-coated surfaces (middle), and 198 pN-coated surfaces (right).

Supplementary Movie 4

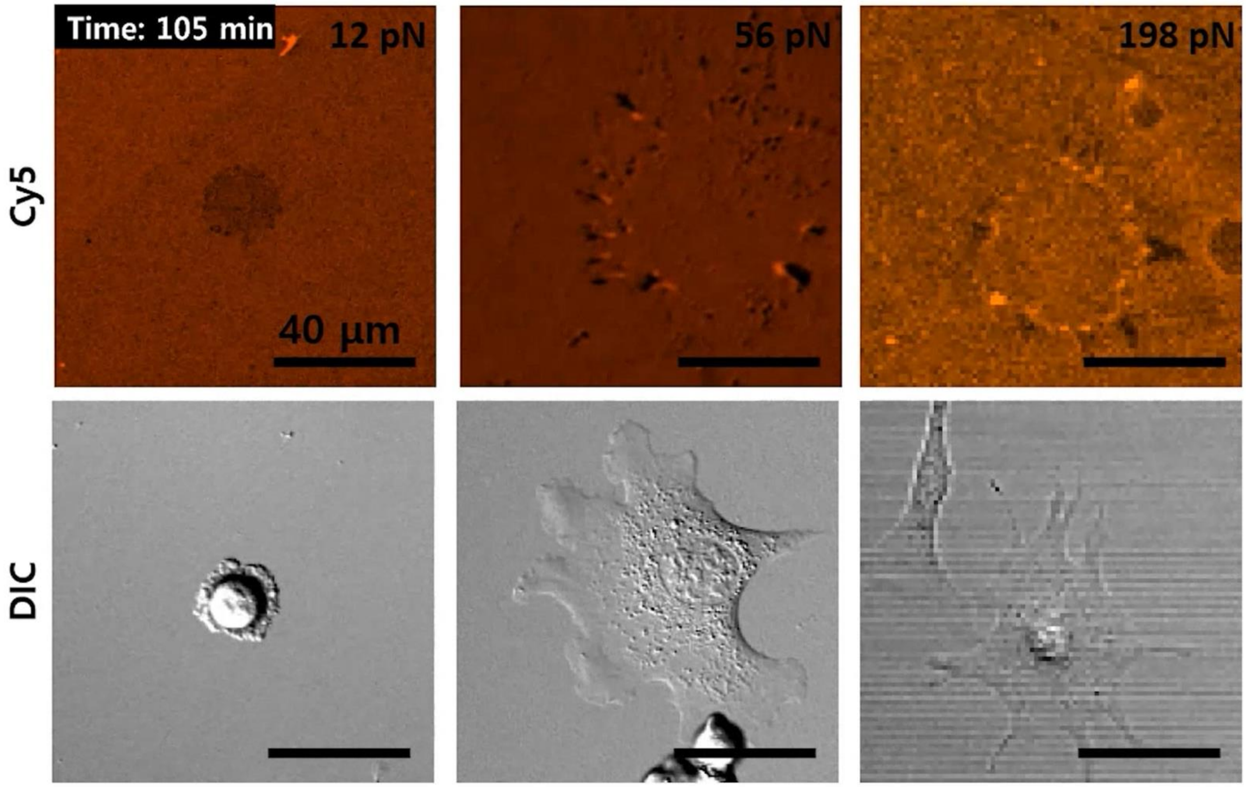

Supplementary Movie 4. Rupture trace of molecular tension probes from FN-coated surfaces. Surfaces were coated with Cy5-conjugated molecular tension probes, which were ruptured by cellular force during cell spreading. The size and number of rupture trace increased as integrin-mediated binding forces were intensified. Tracking images were captured every 10 min for 6 h on 12 pN tension probe-coated surfaces (left), 56 pN-coated surfaces (middle), and 198 pN-coated surfaces (right).

Supplementary Movie 5

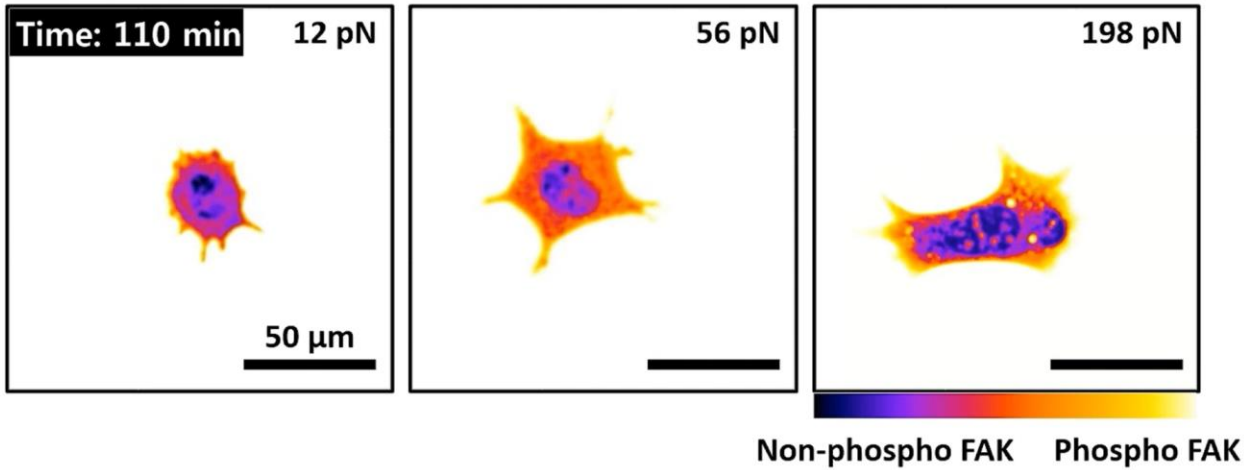

Supplementary Movie 5. Time-lapse monitoring of FAK phosphorylation on molecular tension probe-coated surfaces.

Integrin-mediated molecular binding force-induced FAK phosphorylation was time-lapse monitored every 30 min for 6 h in MEFs on 12 pN tension probe-coated surfaces (left), 56 pN-coated surfaces (middle), and 198 pN-coated surfaces (right). Cells were transfected with cytosolic FAK FRET sensors.

Supplementary Movie 6

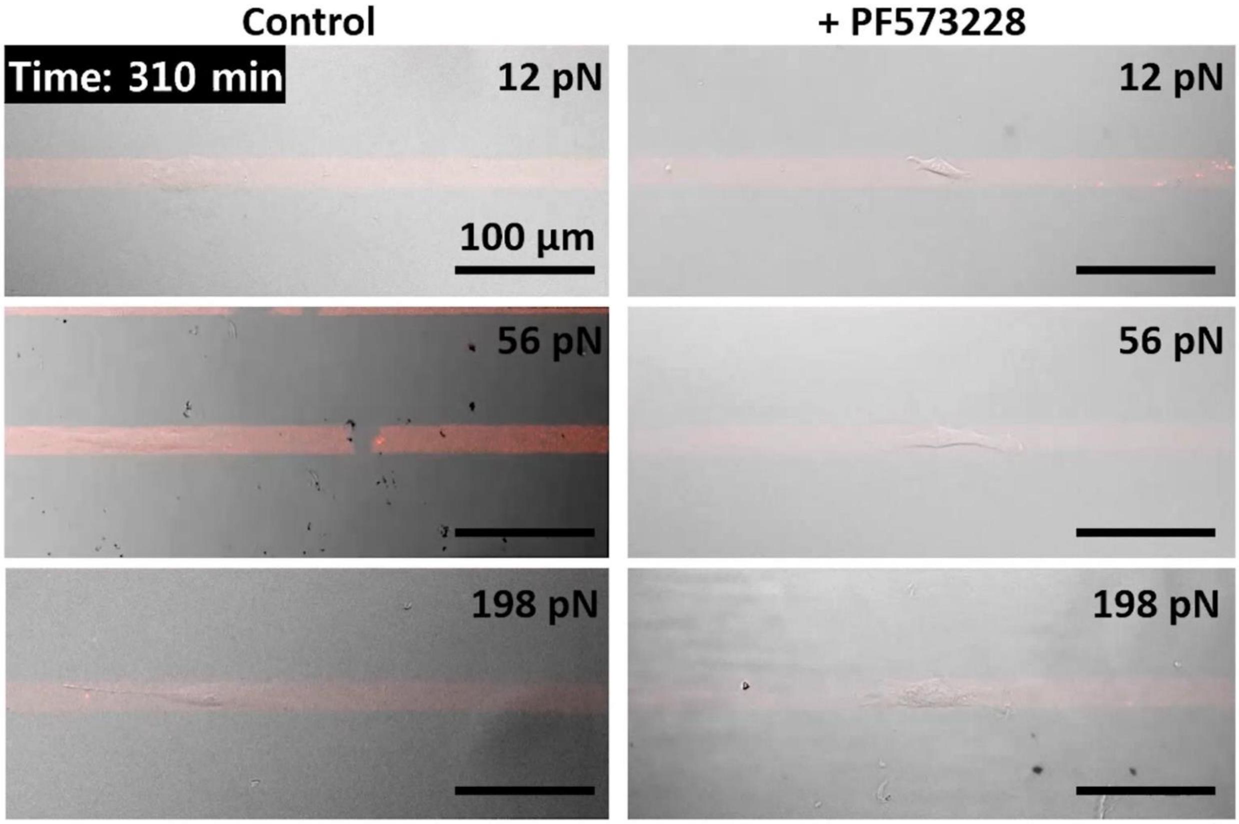

Supplementary Movie 6. Directional migration of MEFs on tension probes-engaged stripe FN-micropatterns.

MEFs plated on tension probe-anchored FN stripes migrated directionally depending on FAK activation controlled by integrin-mediated molecular binding force. Cells were monitored every 10 min for 12 h on 12 pN tension probe-coated surfaces (top), 56 pN-coated surfaces (middle), and 198 pN-coated surfaces (bottom) with/without PF573228 treatment.

Supplementary Movie 7

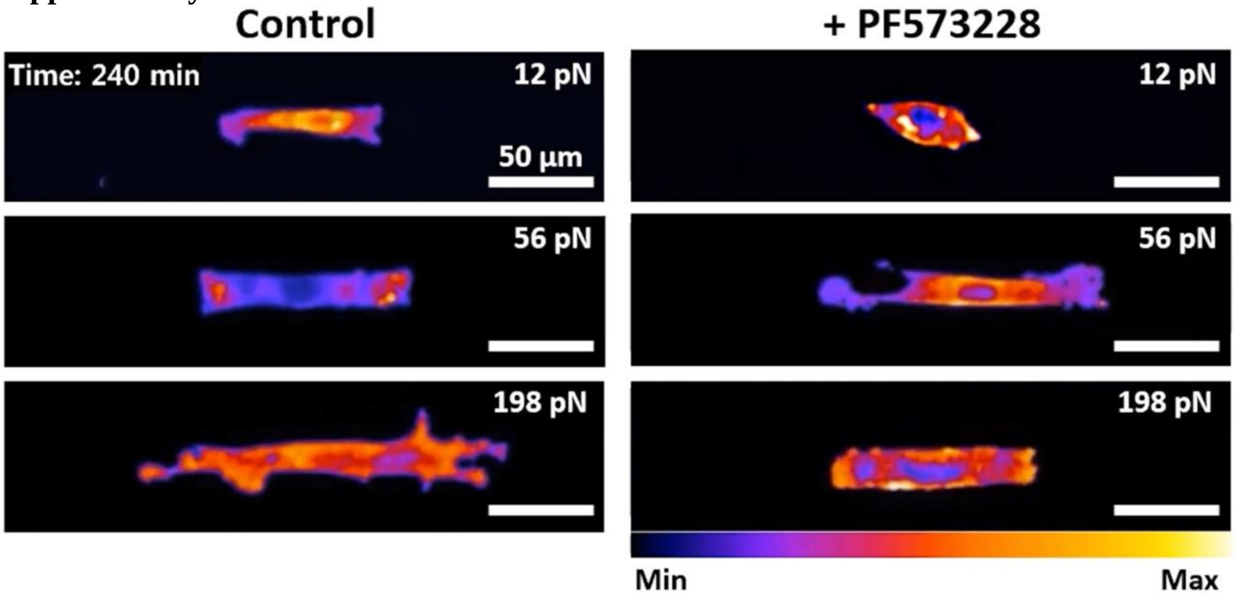

Supplementary Movie 7. Time-lapse monitoring of activated Cdc42 during directional cell migration.

Cdc42 in control MEFs was activated on the front end of cell depending on FAK phosphorylation. Cells transfected with Cdc42 FRET sensors were monitored every 10 min for 12 h on 12 pN tension probe-coated surfaces (top), 56 pN-coated surfaces (middle), and 198 pN-coated surfaces (bottom) with/without PF573228 treatment.

Supplementary Movie 8

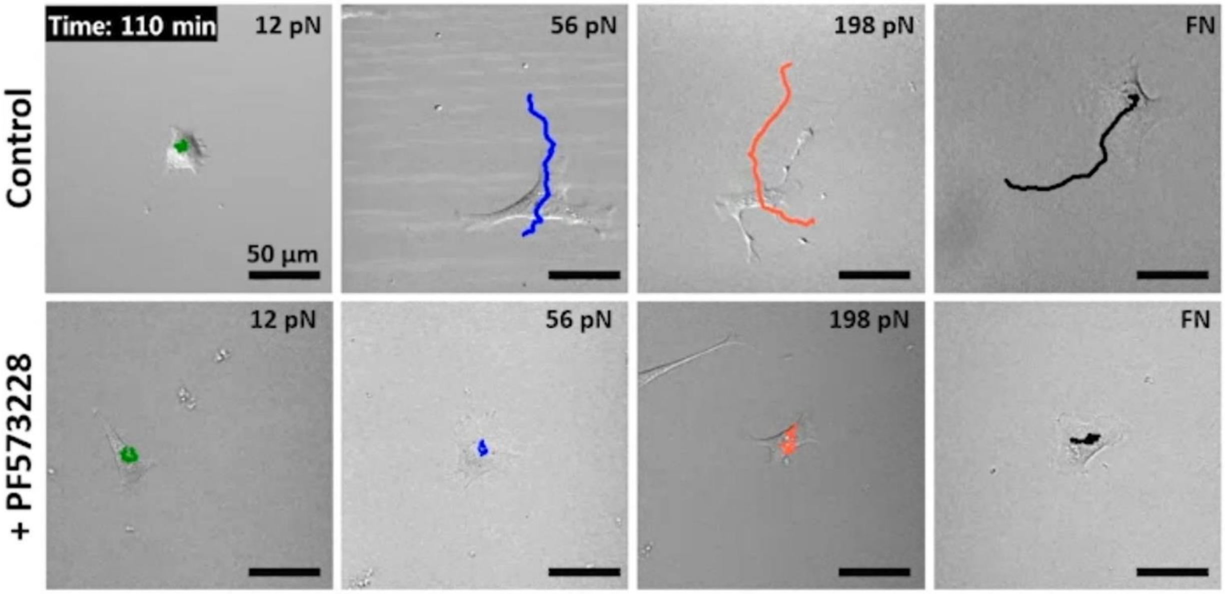

Supplementary Movie 8. Monitoring cell migration on molecular tension probe-coated surfaces.

MEFs placed on 12 pN, 56 pN, and 198 pN tension probes-coated FN surfaces or tension probe-absent FN surface were time-lapse monitored with/without PF573228 treatment. Images were captured every 10 min for 6 h.

Supplementary Movie 9

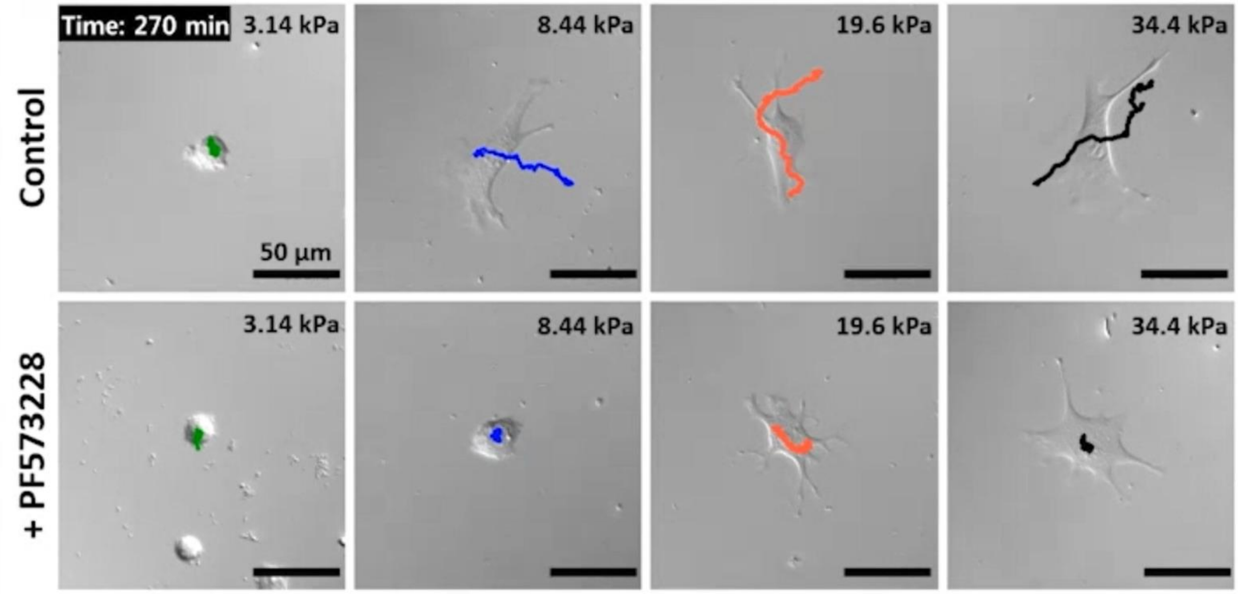

Supplementary Movie 9. Monitoring cell migration on PA-gels with differential rigidity. MEFs placed on PA-gels of varying substrate rigidity from 3.14 kPa to 8.44 kPa, 19.6 kPa, and 34.4 kPa were time-lapse monitored with/without PF573228 treatment. Cell migration images were acquired every 10 min for 6 h.
